# Supplementary material for: Structural basis of lipopolysaccharide assembly by the outer membrane translocon holo-complex
Source: Nat Commun. 2025 Nov 24;16:10404. doi: 10.1038/s41467-025-65370-2 (PMC12644819; doi:10.1038/s41467-025-65370-2)
Supplement: Supplementary file 1 — Supplementary Information [file 41467_2025_65370_MOESM1_ESM.pdf]

SUPPLEMENTARY INFORMATION

Structural Basis of Lipopolysaccharide Assembly by the Outer Membrane Translocon Holo-Complex

Haoxiang Chen, Axel Siroy, Violette Morales, Dominik Gurvic, Yves Quentin, Stephanie Balor, Yassin A. Abuta'a, Maurine Marteau, Carine Froment, Anne Caumont-Sarcos, Julien Marcoux, Phillip J. Stansfeld, Rémi Fronzes, Raffaele Ieva

Supplementary Information includes:

Supplementary Methods

Supplementary Figures 1-19

Supplementary Tables 1-7

Other Supplementary Materials include:

Supplementary Movie

Source Data file

Supplementary Data file

SUPPLEMENTARY METHODS

Genome samples

A representative set of *Enterobacterales* genomes was assembled by selecting genomes at genus (157 genomes) and species level (848 genomes) from the Genome Taxonomy Database (GTDB release 214) [1] (see Source Data file, sheet "GTDB_EnterobacteralesMetadata"), which were downloaded from the NCBI website ftp://ftp.ncbi.nlm.nih.gov/genomes (ftp links listed in Source Data file, sheet "GTDB_EnterobacteralesGenomes"). Genomes were annotated with *Prokka* (version 1.14.6) in fast mode with default settings [2] (see Source Data file, sheet "GTDB_EnterobacteralesAnnotation").

Orthologous gene clusters and species tree

OrthoFinder software (version 2.5.4) [3] was used to identify orthologous gene clusters (OGs) in the 157 representative genomes *of the Enterobacterales* genera. Before inferring the species tree, the 13 genomes with the fastest rates of evolution were discarded. 277 OGs, with zero or one gene copy per genome, were selected. Sequences were aligned with *mafft* (option -M msa) [4]. Alignment sites with a gap frequency greater than 50% were removed and alignments concatenated. These steps were performed by OrthoFinder. The alignment contains 145 concatenated sequences and 80417 sites.

The tree was inferred with *IQ-TREE2* (version 2.1.4-beta) [5] and branch supports were assessed with ultrafast bootstrap approximation and with single branch tests (-*m LG+R10 -B 1000 -bnni -alrt 1000*). The tree was rooted with *Budviciaceae* as an outgroup and displayed with *iTOL* [6]. The *Enterobacterales* family has been divided into seven clades: the *Enterobacteriaceae* clade, and the new families *Erwiniaceae*, *Pectobacteriaceae*, *Yersiniaceae*, *Hafniaceae*, *Morganellaceae* and *Budviciaceae* [7]. In accordance with the species reported in this study, we generated a tree of the species present in our dataset. The branches, especially the deeper ones, are very

well supported by the bootstrap values (Supplementary Fig. 2). The weakest branches are found in a subtree of the *Pectobacteriaceae*, whose leaves present very small genomes. The corresponding genera have no family assigned in the List of Prokaryotic names with Standing in Nomenclature (LPSN) [8] (*Schneideria*, *Baumannia*, *Mikella*, *Gullanella*, *Steffania*, *Hoaglandella*, and *Doolittlea*) or belong to *Enterobacteriaceae* (*Blochmannia* and *Moranella*). We used NCBI's BioSample database to obtain genomes metadata.

Search of proteins candidates

We chose LptDEM proteins as a signature for the LPS transporter in the *Enterobacterales* genomes. Annotation of the protein families were performed with *hmmscan* (*HMMER* package version 3.1b2) [9] and the Pfam library (version 34.0). Selected proteins were scanned against the Pfam library using *hmmscan* to discard false positives. Results were filtered to retain only alignments covering at least 75% of the Pfam domain and an independent E-value (i-Evalue) < 1e-15 for all but LptE and LptM (i-Evalue < 1e-5). Only one occurrence was retained per profile and per genome.

LptD is covered by two Pfam profiles (PF04453 and PF03968). The profile PF03968 is shared with LptA. Using a threshold of 50% for the percentage of profile coverage on the protein, we were able to distinguish between LptA and LptD proteins.

As previously observed [10], genes encoding the short lipoprotein LptM were not always annotated by *Prokka*. To complete the annotation, genomes for which no LptM had been identified were translated in all six reading phases with *esl-translate* program from *HMMER* package. *hmmsearch* was used to identify the presence of the PF13627 domain in translated ORFs (trORF). As above, *hmmscan* was used against the retained trORFs to remove false positives.

E. coli (strain K12) LptY (YEDD_ECOLI) is a lipoprotein of 137 amino acids long. LptY is characterized by Pfam PF13987. 359 and 58 sequences are selected from

species and genus genome samples, respectively. We used the NCBI web server to search for YEDD_ECOLI in non-redundant protein sequences, excluding *Enterobacterales*. Both BlastP and BlastX software were used. The results show a small number of sequences (43) with high similarity to *E. coli* LptY. We aligned these sequences with those obtained from the *Enterobacterales* in our sample and inferred a tree. The non-*Enterobacterales* LptY proteins are identical or very close to *Enterobacterales* sequences and are generally partial. These observations indicate that these sequences are the result of either recent horizontal transfer or, more likely, sequencing artifacts.

LptY tree

The 58 reference LptY protein sequences at genus level were aligned with *mafft* (--localpair --maxiterate 1000) [4]. The filtered alignment with *trimAl* (-gappyout) included 136 amino acid sites [11]. The LG4M substitution model was selected using *ModelFinder* [12]. The tree (Supplementary Fig. 3) was inferred with *IQ-TREE2* [5] and branch supports were estimated with ultra-fast bootstrap approximation, single branch tests (*-B 1000 -bnni -alrt 1000*) and standard non-parametric bootstrap (*-b 100*).

The genomic context of *lptY* gene candidates was extracted from GFF3 Prokka annotation files over a window of 5000 nucleotides upstream and 10000 nucleotides downstream of these genes. The genes are associated with their HOG to illustrate the conservation of *lptY*'s genetic context. Pfam profiles associated with these HOGs were identified with *hmmscan* as described above.

Analysis of *Enterobacterales* species tree

The tree is consistent with the new classification of the *Enterobacterales* family into seven clades [7]: *Enterobacteriaceae*, *Erwiniaceae*, *Pectobacteriaceae*, *Yersiniaceae*, *Hafniaceae*, *Morganellaceae* and *Budviciaceae*; the branches, especially the deeper ones, are very well supported by the bootstrap values (Supplementary Fig. 2). The

tree has been generated with genomes representing genera, but each genus may contain several genomes in our dataset (Supplementary Fig. 2, grey histogram). Intra-genus variability is illustrated by the size of the symbol, which is proportional to the frequency with which the feature is observed in the genomes of that genus. Genomes with significant genome reduction (Supplementary Fig. 2, genome size column) have accelerated sequence evolution, resulting in long branches. They almost always have arthropods as hosts, often in symbiotic associations (Supplementary Fig. 2, Metadata). The *Enterobacteriaceae* family includes almost all vertebrate pathogens but with low frequencies (Supplementary Fig. 2, Metadata, small blue squares), which may reflect the poor metadata annotation of these genomes. The LptY protein has been identified in the *Enterobacteriaceae* and *Erwiniaceae* families, and with less frequency in the *Pectobacteriaceae* family. In *Erwiniaceae*, its absence is accompanied by the absence of other OM LPS translocon protein partners. In the *Pectobacteriaceae*, the *lptY* gene is absent in genomes from the first clade encompassing pathogens of flowering plants (*Magnoliopsida*) and has a dispersed distribution in the second clade whose bacteria often have arthropods as hosts. The tree obtained with LptY (Supplementary Fig. 3) is in agreement with the species tree (Supplementary Fig. 2), therefore it does not support acquisition of the LptY gene by horizontal transfer but rather suggests that the absence of this gene in certain species is due to independent loss events. Overall, our analyses are consistent with a recent acquisition of the *lptY* gene in the last common ancestor of the *Pectobacteriaceae*, *Erwiniaceae* end *Enterobacteriaceae* (Supplementary Fig. 2, blue dot) followed by multiple independent losses of this gene (Supplementary Fig. 2, red dots) and to a lesser extent of *lptM, lptE* and *lptD* in species that have undergone a drastic reduction in genome size, such as the endosymbionts.

In-gel tryptic digestion

LptDEM^{His} and LptDE^{His} complexes purified for cryo-EM experiments were loaded

and separated on 4-12% polyacrylamide gradient gel (Merck) using MES buffer. Protein bands were revealed by Coomassie Brilliant Blue staining. Bands of interest were excised from the gel, cut into small pieces to perform in-gel digestion as previously described [13]. After several washing steps to eliminate stain, the pieces of gel were dried under vacuum. Proteins were reduced with 10 mM DTT in 100 mM ammonium bicarbonate buffer for 35 min at 56 °C and then alkylated with 55 mM iodoacetamide for 30 min at room temperature in the dark. The gel fragments were dried under vacuum and swollen in a covering volume of modified trypsin (Promega, Madison, WI, USA) solution (12.5 ng/ml in 50 mM ammonium bicarbonate buffer) for 15 min in an ice bath followed by overnight incubation at 37 °C. The supernatant was pooled with two peptide extracts performed at 37°C for 15 min with shaking in 5% formic acid in 50% acetonitrile. The peptide mixture was dried down with the speed vacuum concentrator and re-suspended in 20 µL of 0.05 % formic acid in 2 % acetonitrile for nano-LC-MS/MS analysis.

LC-MS/MS analysis

Samples were analysed using an Ultimate 3000 nanoRS system coupled to a Q-Exactive Plus mass spectrometer (Thermo Fisher Scientific, Bremen, Germany) operating in positive mode. 5 µL of each sample was loaded onto a C18-precolumn (300 µm inner diameter x 5 mm) at 20 µL/min in 2% ACN, 0.05% trifluoroacetic acid (TFA). After 5 min of desalting, the precolumn was switched online with the analytical C18 column (75 µm inner diameter × 50 cm, in-house packed with Reprosil C18) equilibrated in 95% solvent A (5% ACN, 0.2% FA) and 5% solvent B (80% ACN, 0.2% FA). Peptides were eluted by using a 5–25% gradient of solvent B for 40 min, then a 25–50% of solvent B for 20 min at a flow rate of 300 nL/min. The Q-Exactive Plus was operated in data-dependent acquisition mode. Survey scans MS were acquired in the Orbitrap over 300–2,000 m/z with a resolution of 70,000 (at m/z 400) an automatic gain control (AGC) target value of 3e6, and a maximum injection time of 100ms. The 10 most intense multiply charged ions (up to 2+) were selected at 2 m/z

and fragmented by Higher Energy Collisional Dissociation (normalized collision energy set to 27). The resulting fragments were analysed in the Orbitrap with a resolution of 17,500 (at 400 m/z), an automatic gain control (AGC) target value of 1e5 and a maximum injection time of 50ms. Dynamic exclusion was used within 30 s with a 10 ppm tolerance, to prevent repetitive selection of the same peptide. For internal calibration, the 445.120025 ion was used as lock mass.

Bioinformatic MS data analysis

Acquired MS and MS/MS data as raw MS files were converted to the mzDB format [14] using the pwiz-mzdb converter (version 0.9.10, https://github.com/mzdb/pwiz-mzdb) executed with its default parameters. Generated mzDB files were processed with the mzdb-access library (version 0.7, https://github.com/mzdb/mzdb-access) to generate peaklists. Peak lists were searched against SwissProt protein database with taxonomy *Escherichia coli* (23,135 sequences) in Mascot search engine (version 2.8.3, Matrix Science, London, UK). Cysteine carbamidomethylation was set as a fixed modification. Methionine oxidation and acetylation of protein N-terminus were set as variable modification. Up to two missed trypsin/P cleavages were allowed. Mass tolerances in MS and MS/MS were set to 10 ppm and 20 mmu, respectively. Proline software [15] was used for the validation and the label-free quantification of identified proteins in each sample. Mascot identification results were imported into Proline. Search results were validated with a peptide rank=1 and at 1 % FDR both at PSM level (on Adjusted e-Value criterion) and protein sets level (on Modified Mudpit score criterion). Label-free quantification was performed for all proteins identified: peptides were quantified by extraction of MS signals in the corresponding raw files, and post-processing steps were applied to filter, normalize, and compute protein intensities. The cross-assignment of MS/MS information between runs was enabled (it allows to assign peptide sequences to detected but non-identified features). Each protein intensity was based on the sum of unique peptide intensities and was normalized across all samples by the median intensity. Protein abundances were

summarized in iBAQ values by dividing the protein intensities by the number of observable peptides in order to determine the protein stoichiometry [16,17]. See also the LC-MS/MS source data in Source Data file.

SUPPLEMENTARY FIGURES AND LEGENDS

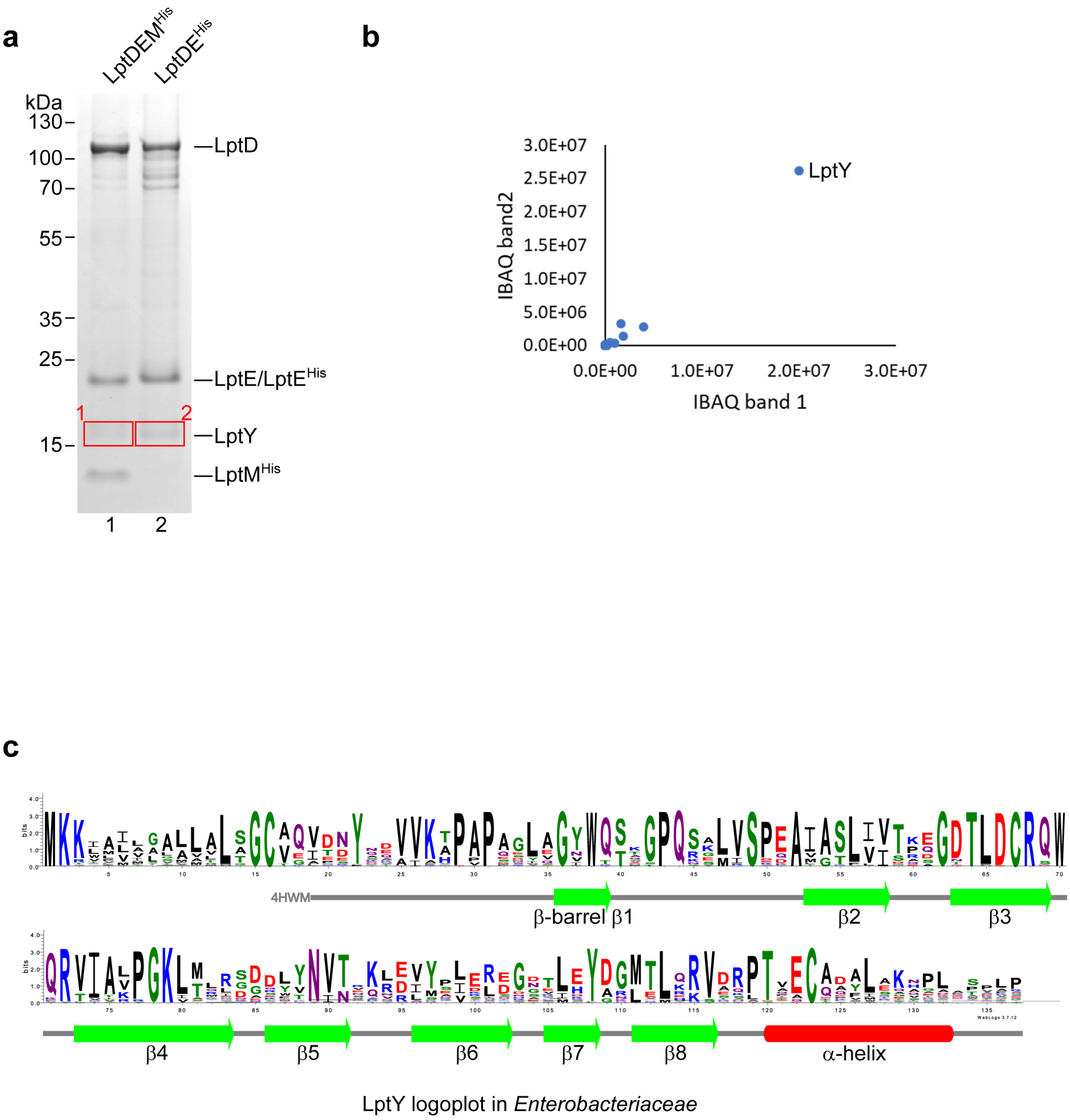

Supplementary Fig. 1. SDS-PAGE based, label-free bottom-up proteomic analysis of LPS translocon complexes

a, LptDEM^{His} and LptDE^{His} samples were prepared as in Fig. 1b, respectively purified from wild-type cells transformed with pLptDEM^{His} and Δ*lptM* cells transformed with pLptDE^{His}. Proteins were separated on SDS PAGE. Gel bands running with an apparent molecular weight of approximately 15 kDa (red boxes) were excised and

their protein contents were analysed by bottom-up mass-spectrometry. **b**, The plot represents iBAQs (intensity-based absolute quantification) of the excised band 1 (iBAQ1, LptDEM^{His}) vs. excised band 2 (iBAQ2, LptDE^{His}). The iBAQ value is obtained by dividing protein intensities by the number of theoretically observable tryptic peptides (see Supplementary Methods). This analysis clearly identifies LptY as the main protein present in the excised band, in both LptDEM^{His} and LptDE^{His} purified complexes. Source data are provided as a Source Data file. **c**, Logoplot of LptY amino acid sequence. The logoplot is based on the alignment of the LptY sequences of the genus reference genomes of *Enterobacteriaceae*.

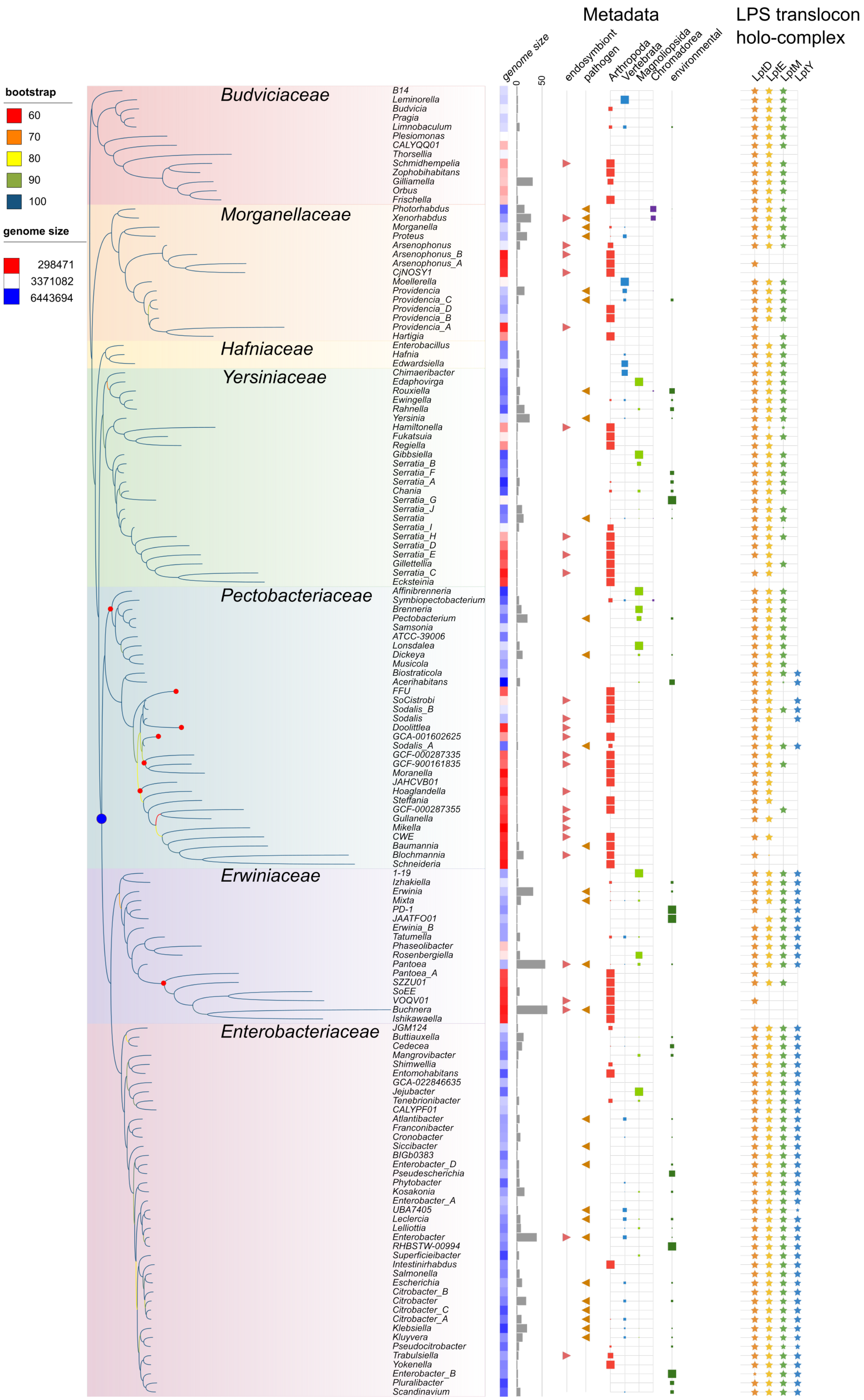

bootstrap
60
70
80
90
100
genome size
298471
3371082
6443694
Budviciaceae
Morganellaceae
Hafniaceae
Yersiniaceae
Pectobacteriaceae
Erwiniaceae
Enterobacteriaceae
genome size
0
50
Metadata
endosymbiont
pathogen
Arthropoda
Vertebrata
Magnoliopsida
Chromadorea
environmental
LPS translocon holo-complex
LptD
LptE
LptM
LptY
B14
Leminorella
Budvicia
Pragia
Limnobaculum
Plesiomonas
CALYQQ01
Thorsellia
Schmidhempelia
Zophobihabitans
Gilliamella
Orbus
Frischella
Photorhabdus
Xenorhabdus
Morganella
Proteus
Arsenophonus
Arsenophonus_B
Arsenophonus_A
CjNOSY1
Moellerella
Providencia
Providencia_C
Providencia_D
Providencia_B
Providencia_A
Hartigia
Enterobacillus
Hafnia
Edwardsiella
Chimaeribacter
Edaphovirga
Rouxiella
Ewingella
Rahnella
Yersinia
Hamiltonella
Fukatsuia
Regiella
Gibbsiella
Serratia_B
Serratia_F
Serratia_A
Chania
Serratia_G
Serratia_J
Serratia
Serratia_I
Serratia_H
Serratia_D
Serratia_E
Gillettellia
Serratia_C
Ecksteinia
Affinibrenneria
Symbiopectobacterium
Brenneria
Pectobacterium
Samsonia
ATCC-39006
Lonsdalea
Dickeya
Musicola
Biostraticola
Acerihabitans
FFU
SoCistrobi
Sodalis_B
Sodalis
Doolittlea
GCA-001602625
Sodalis_A
GCF-000287335
GCF-900161835
Moranella
JAHCVB01
Hoaglandella
Steffania
GCF-000287355
Gullanella
Mikella
CWE
Baumannia
Blochmannia
Schneideria
1-19
Izhakiella
Erwinia
Mixta
PD-1
JAATFO01
Erwinia_B
Tatumella
Phaseolibacter
Rosenbergiella
Pantoea
Pantoea_A
SZZU01
SoEE
VOQV01
Buchnera
Ishikawaella
JGM124
Buttiauxella
Cedecea
Mangrovibacter
Shimwellia
Entomohabitans
GCA-022846635
Jejubacter
Tenebrionibacter
CALYPF01
Atlantibacter
Franconibacter
Cronobacter
Siccibacter
BIGb0383
Enterobacter_D
Pseudescherichia
Phytobacter
Kosakonia
Enterobacter_A
UBA7405
Leclercia
Lelliottia
Enterobacter
RHBSTW-00994
Superficieibacter
Intestinirhabdus
Salmonella
Escherichia
Citrobacter_B
Citrobacter
Citrobacter_C
Citrobacter_A
Klebsiella
Kluyvera
Pseudocitrobacter
Trabulsiella
Yokenella
Enterobacter_B
Pluralibacter
Scandinavium

Supplementary Fig. 2. Distribution of LptY proteins in *Enterobacterales* in relation to genome features and the LPS transporter

We used a single representative genome per genus of *Enterobacterales*. The phylogenetic tree inferred with *IQ-TREE2* (Minh et al. 2020) on an alignment of 145 concatenated sequences and 80417 sites. The lengths of the branches are proportional to the distances inferred by *IQ-TREE2*. The branch supports, reported as branch color gradient, were assessed with the SH-like approximate likelihood ratio test (-alrt 1000). The tree was rooted with *Budviciaceae* family. *Enterobacterales* are divided in seven families. Columns annotations, genome size: color gradient of the genome sizes with small genomes in red and large genomes in blue, grey histogram: number of genomes in each genus, Metadata: metadata extracted from NCBI's BioSample database, LPS translocon holo-complex: LptDEMY proteins. Intra-genus variability is illustrated by the size of the symbol, which is proportional to the frequency with which the feature is observed in the genomes of that genus. The figure has been generated using iTOL (see Supplementary Methods). The blue dot on the tree represents the node where the lptY gene is thought to have been acquired, and the red dots indicate several independent losses of this gene.

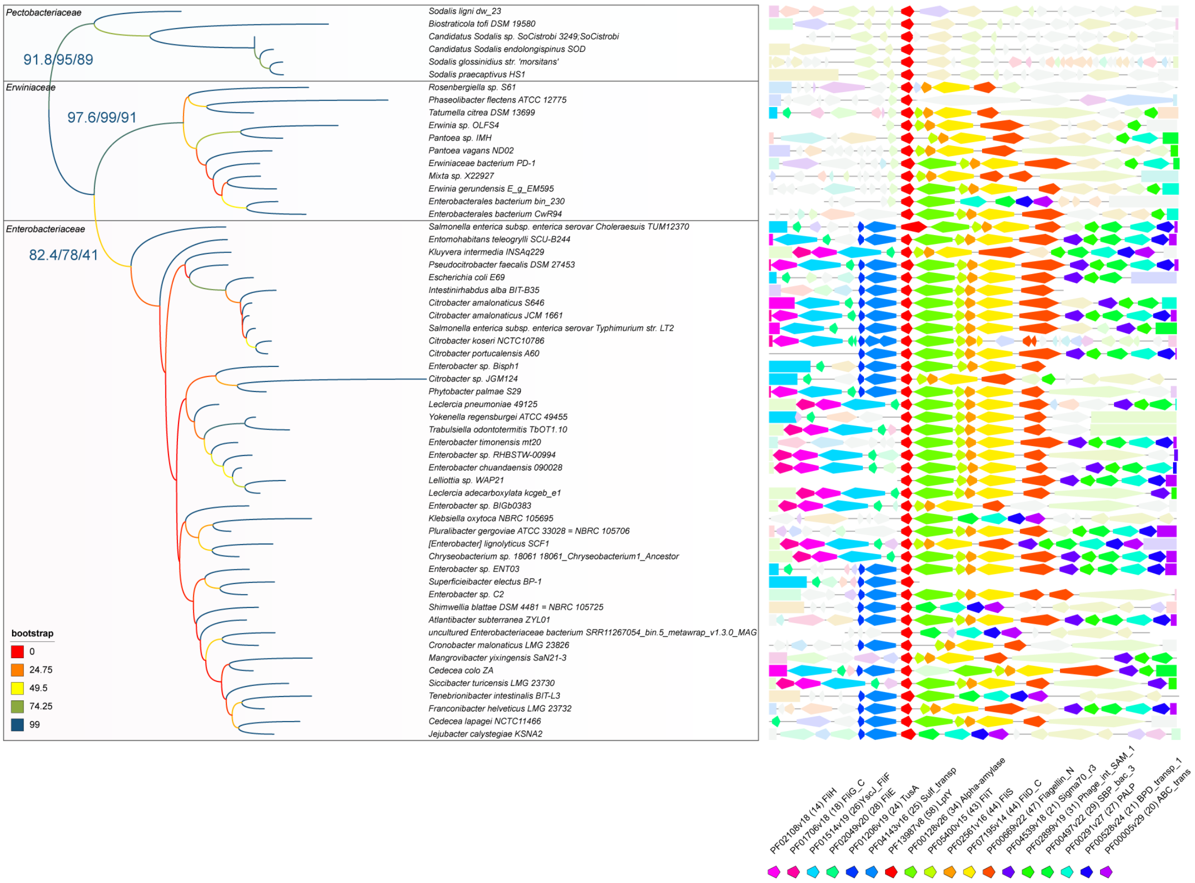

Supplementary Fig. 3. LptY protein tree in the *Enterobacterales* genera

The tree was inferred with *IQ-TREE2* (see Supplementary Methods), LptY sequences are provided as a Supplementary Data file. Branch supports were estimated using the ultra-fast bootstrap approximation, the SH approximate likelihood ratio test and the non-parametric bootstrap. The values obtained were displayed on the three deepest branches of the tree and the non-parametric bootstrap were plotted on the tree as a gradient of branch color. The division of *Enterobacterales* into three families was reported. The genomic contexts of the *lptY* genes extracted 5000 nucleotides upstream and 10000 nucleotides downstream of the *lptY* genes were displayed. A colour code was used to highlight genes sharing the same Pfam domain annotation. Domain accession, name and frequency are indicated. Very light color were used for genes present in fewer than 14 neighbourhoods. The figure has been generated using iTOL (see Supplementary Methods). Fasta file is provided as Supplementary Data file.

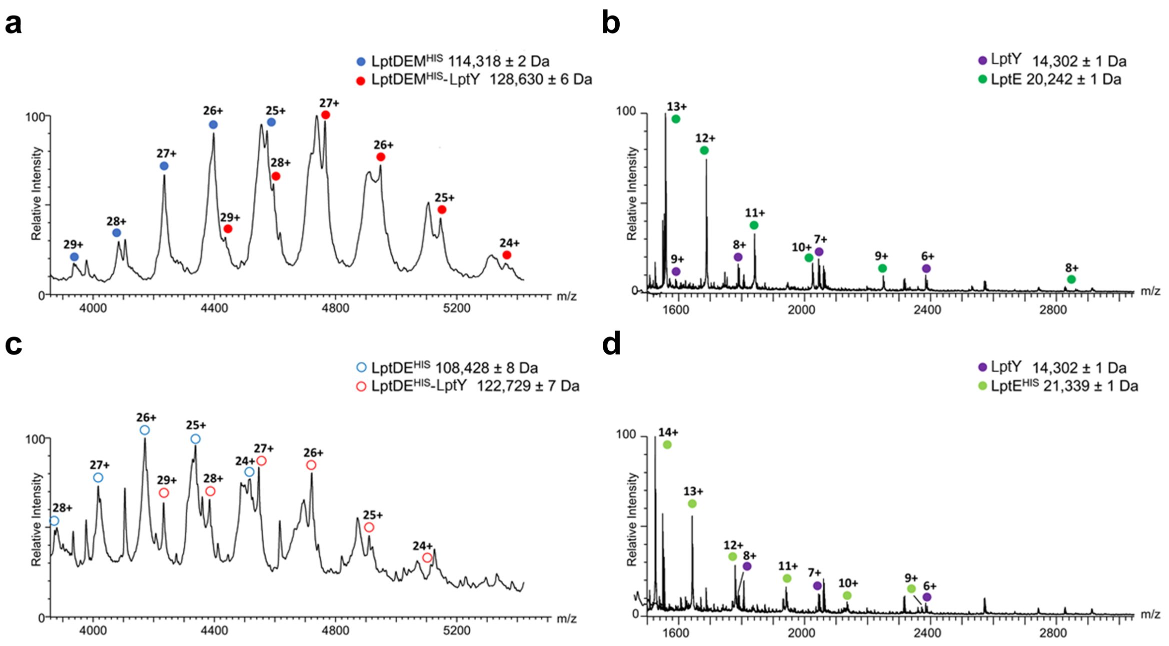

Supplementary Fig. 4. Native MS of the LPS translocons

Mass spectra of (**a**) LptDEM^{His} and (**b**) LptDE^{His} aquired under conditions to maintain non-covalent interactions shows the presence of the corresponding translocons alone and with a mass shift of of 14,3 kDa. A gas-phase dissociated monomer with MW=14,302 Da, corresponding to LptY can be observed in the lower m/z region for both the (**c**) LptDEM^{His} and (**d**) LptDE^{His} complexes.

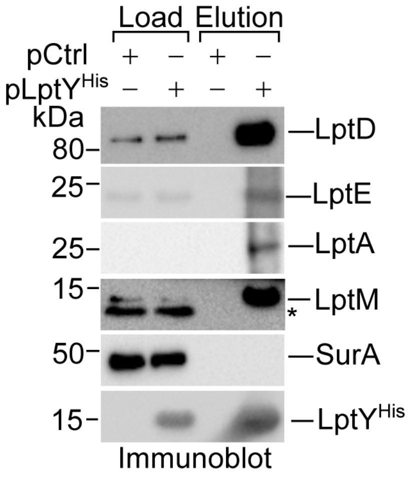

Supplementary Fig. 5. **Pull down of LptY co-isolates components of the LPS OM translocon and LptA**

Δ*lptY* transformed with the empty vector pCtrl or with pLptY^{His} were subjected to envelope fractionation, solubilization with DDM and nickel-affinity purification of LptY^{His}. Imidazole eluted proteins were subjected to SDS-PAGE and revealed by immunoblotting using the indicated antisera or monoclonal anti-His antibodies to visualize LptY. Load: 0.5%; Elution 100%; * indicates a non-specific reaction. The result is representative of three independent experimental repeats.

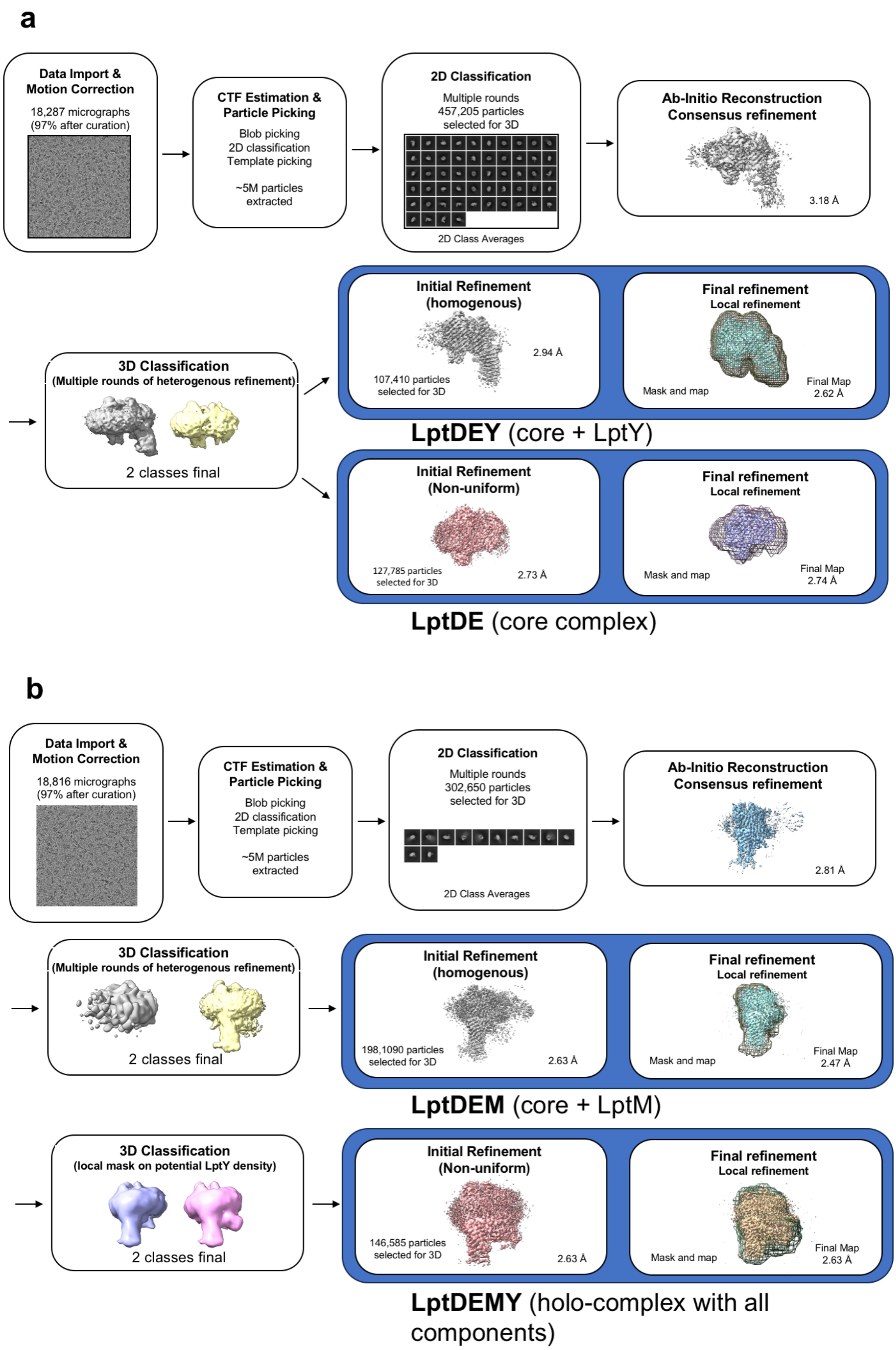
a
Data Import & Motion Correction
18,287 micrographs (97% after curation)
CTF Estimation & Particle Picking
Blob picking
2D classification
Template picking
~5M particles extracted
2D Classification
Multiple rounds
457,205 particles selected for 3D
2D Class Averages
Ab-Initio Reconstruction
Consensus refinement
3.18 Å
3D Classification
(Multiple rounds of heterogenous refinement)
2 classes final
Initial Refinement (homogenous)
2.94 Å
107,410 particles selected for 3D
Final refinement
Local refinement
Mask and map
Final Map 2.62 Å
LptDEY (core + LptY)
Initial Refinement (Non-uniform)
127,785 particles selected for 3D
2.73 Å
Final refinement
Local refinement
Mask and map
Final Map 2.74 Å
LptDE (core complex)
b
Data Import & Motion Correction
18,816 micrographs (97% after curation)
CTF Estimation & Particle Picking
Blob picking
2D classification
Template picking
~5M particles extracted
2D Classification
Multiple rounds
302,650 particles selected for 3D
2D Class Averages
Ab-Initio Reconstruction
Consensus refinement
2.81 Å
3D Classification
(Multiple rounds of heterogenous refinement)
2 classes final
Initial Refinement (homogenous)
198,1090 particles selected for 3D
2.63 Å
Final refinement
Local refinement
Mask and map
Final Map 2.47 Å
LptDEM (core + LptM)
3D Classification
(local mask on potential LptY density)
2 classes final
Initial Refinement (Non-uniform)
146,585 particles selected for 3D
2.63 Å
Final refinement
Local refinement
Mask and map
Final Map 2.63 Å
LptDEMY (holo-complex with all components)

Supplementary Fig. 6. CryoSPARC single particle analysis workflow for LPS translocon complexes.

CryoSPARC processing workflows for two datasets yielding four final maps: (**a**) Dataset 1 generating LptDEY complex (LptDE core + LptY, 2.74 Å) and LptDE core complex (2.62 Å), and (**b**) Dataset 2 generating LptDEM complex (LptDE core + LptM, 2.47 Å) and LptDEMY holo-complex (complete translocon, 2.63 Å). Each workflow shows the progression from data import and motion correction through 2D/3D classification to final map refinement. Representative micrographs, 2D class averages, 3D reconstructions from key processing steps (3D classification, intermediate and final refinements), and particle counts are indicated. Detailed procedures are described in the Methods section.

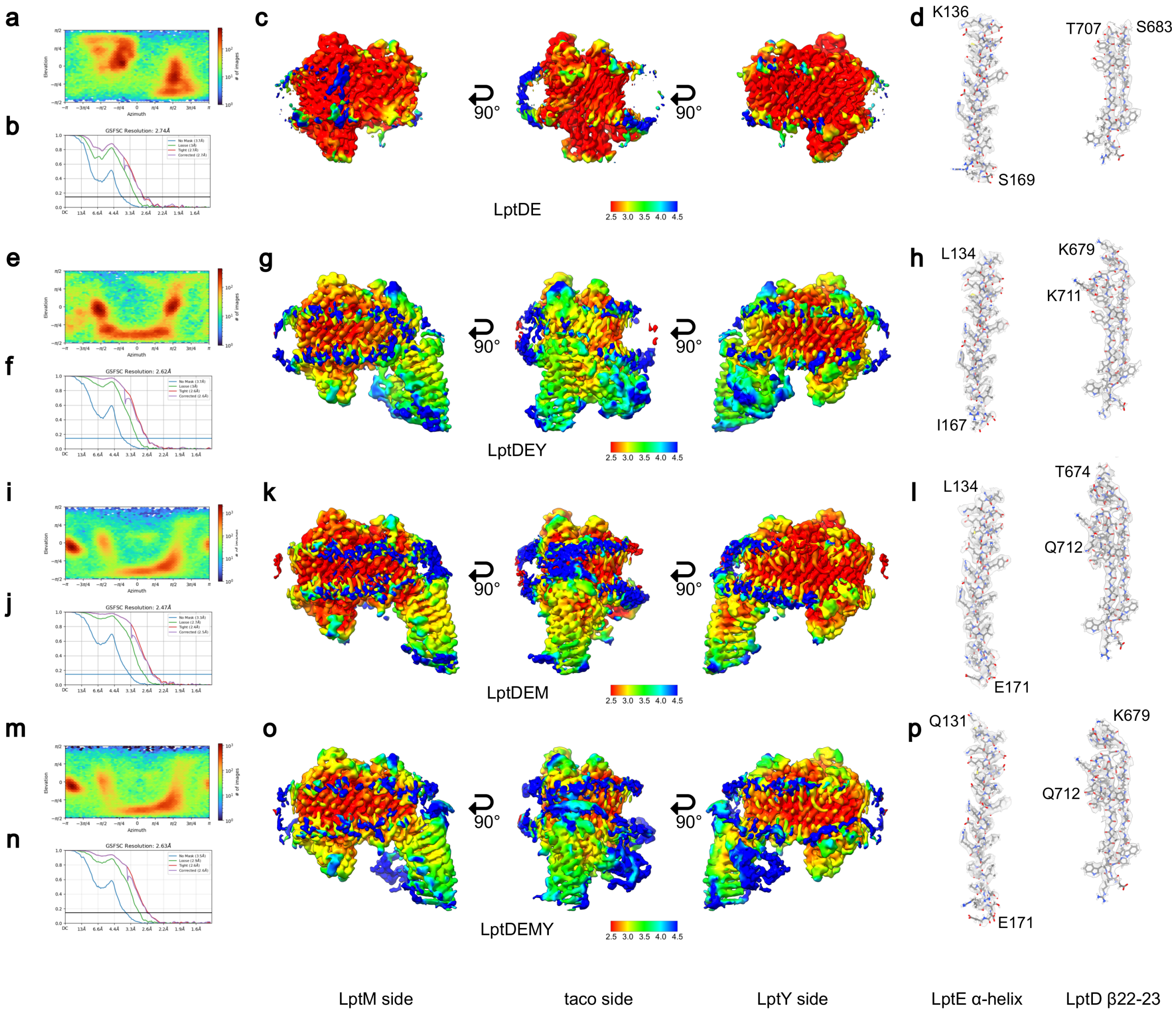

Supplementary Fig. 7. Local refinement of the LptDE heterocomplexes

a, Particle orientation distribution of the LptDE heterodimer, obtained in Cryosparc. **b,** Fourrier Shell Correlation (FSC) map obtained from half-maps in Cryosparc. **c,** Local-filtered map of LptDE, at level 0.219, coloured by local resolution. **d,** Fitting of the structural model into the cryo-EM density map: the C-terminal α-helix of LptE and the β-strands 22 and 23 from LptD β-barrel. **e,** Particle orientation distribution of the LptDE-LptY oligomer, obtained in Cryosparc. **f,** Fourrier Shell Correlation (FSC) map obtained from half-maps in Cryosparc. **g,** Local-filtered map of LptDEY, at level 0.168, coloured by local resolution. **h,** Fitting of the structural model into the cryo-EM density map. **i,** Particle orientation distribution of the LptDEM complex, obtained in Cryosparc. **j,** Fourrier Shell Correlation (FSC) map obtained from half-maps in

Cryosparc. **k,** Local-filtered map of LptDEM, at level 0.161, coloured by local resolution. **l,** Fitting of the structural model into the cryo-EM density map. **m,** Particle orientation distribution of the LptDEMY complex, obtained in Cryosparc. **n,** Fourrier Shell Correlation (FSC) map obtained from half-maps in Cryosparc. **o,** Local-filtered map of LptDEMY, at level 0.171, coloured by local resolution. **p,** Fitting of the structural model into the cryo-EM density map.

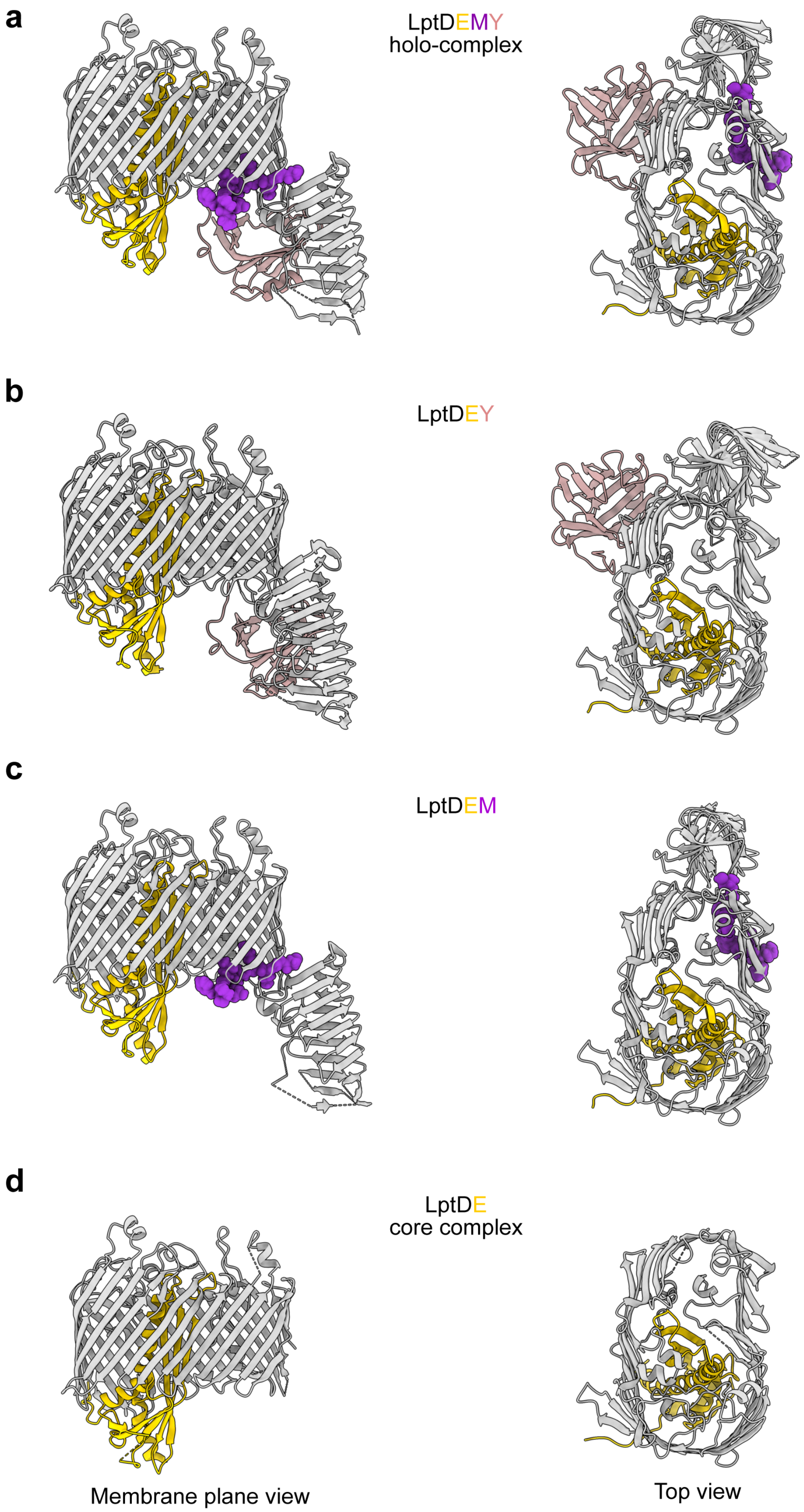
a
LptdDEMY
holo-complex
b
LptDEY
c
LptDEM
d
LptDE
core complex
Membrane plane view
Top view

Supplementary Fig. 8. Views of the resolved structure

Ribbon representations of the resolved structures seen from the membrane plane (**letf**) and from the top (**right**) with LptD in grey, LptE in yellow, LptM in purple and LptY in pink. **a**, LptDEMY holo-complex **b,** LptDEY complex. **c**, LptDEM complex. **d**, LptDE core complex.

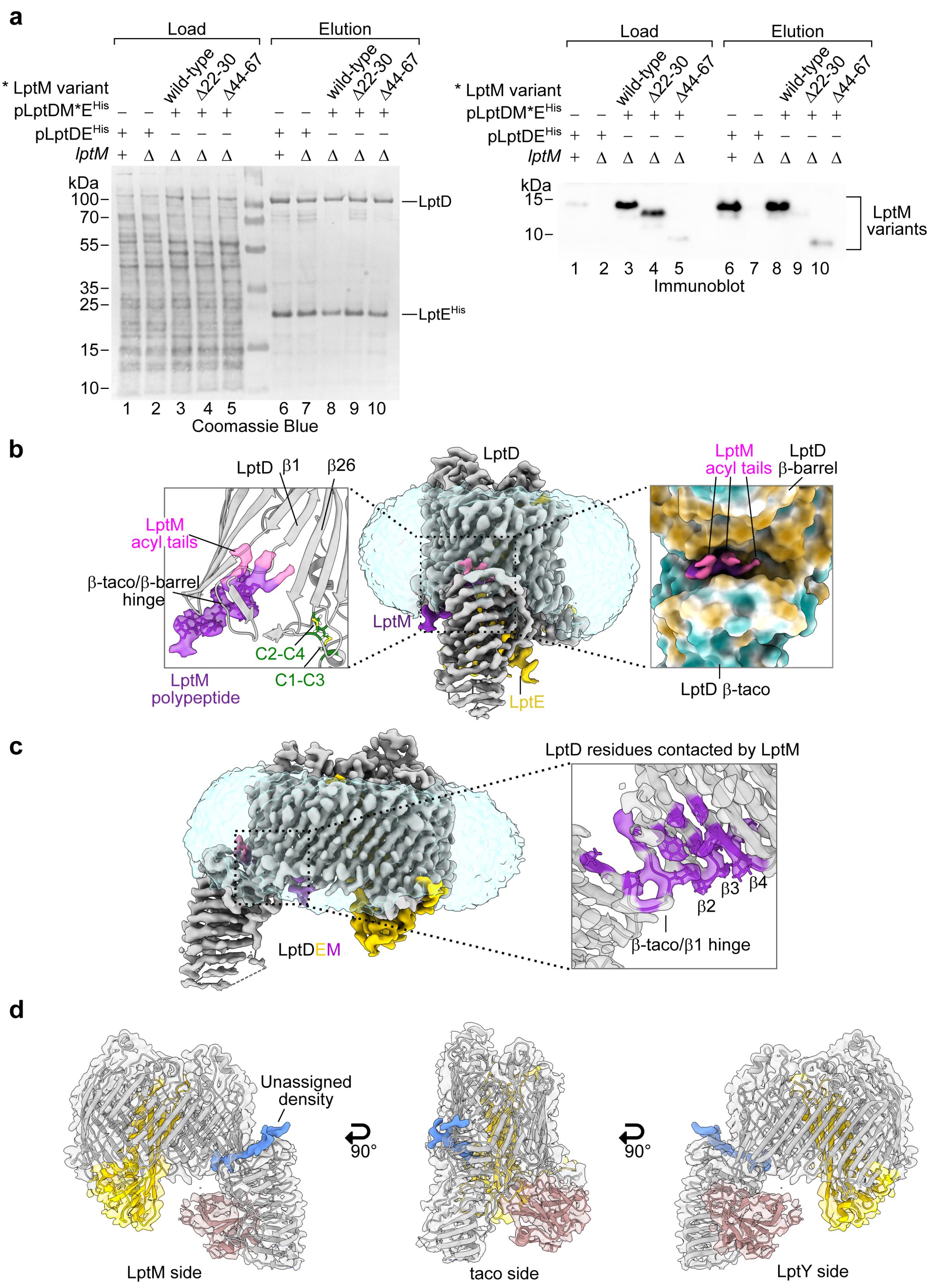

a
Load
Elution
* LptM variant
wild-type
Δ22-30
Δ44-67
pLptDM*E^{His}
pLptDE^{His}
lptM
kDa
100
70
55
35
25
15
10
LptD
LptE^{His}
1 2 3 4 5 6 7 8 9 10
Coomassie Blue
LptM variants
Immunoblot
b
LptD β1 β26
LptM acyl tails
β-taco/β-barrel hinge
C2-C4
C1-C3
LptM polypeptide
LptD
LptM
LptE
LptD β-barrel
LptD β-taco
c
LptD residues contacted by LptM
LptDEM
β2 β3 β4
β-taco/β1 hinge
d
Unassigned density
90°
LptM side
taco side
LptY side

Supplementary Fig. 9. The N-terminal portion of LptM interacts with the translocon

a, Wild-type or Δ*lptM* cells transformed with the indicated plasmids were subjected to envelope fractionation, solubilization with a mild detergent and nickel-affinity purification of $LptE^{His}$. Only the LptM variants containing the protein N-terminal portion (wild-type LptM and $LptM^{\Delta 44\text{-}67}$) were enriched in the elution fractions. Load: 1%; Elution 100%. The result is representative of three independent experimental repeats. **b, Left**, Zoom on LptM in the structure of LptDEM complex, highlighting the electron density corresponding to the acyl tails of LptM (pink) and the electron density corresponding to LptM N-terminal moiety (purple) situated near the lateral gate of LptD (β1- β26) and between the β-taco/ β-barrel hinge and the LptD inter-domain disulfide bonds. LptD Cys31 (C1), Cys173 (C2), Cys724 (C3) and Cys725 (C4) are shown in green with disulfide bonds in yellow. **Central**, cryo-EM map of the LptDEM complex coloured according to the protein chains (LptD in grey, LptE in yellow, LptM in purple). **Right**, zoom on the lipid tails of LptM and the surrounding surface of LptD coloured based on hydrophobicity of the amino acid side chains (brown, hydrophobic; cyan, hydrophilic). **c, Left**, cryo-EM map of the LptDEM complex coloured according to the protein chain (LptD in grey, LptE in yellow, LptM in purple). **Right**, internal section of the LptD β-taco/β-barrel hinge region. The LptD residues in contact with LptM are shown in purple. **d,** Cryo-EM density map of the *E. coli* LptDEY (LptDE-YedD) complex (PDB: 9FZ5) [18] showing the lateral gate region from multiple orthogonal side views. The LptD β-barrel (gray), LptE (yellow), and LptY (pink) are shown as cartoon representations. An unassigned extra density (blue density) is observed in a position similar to where LptM binds in our LPS translocon structures. This extra density could correspond to LptM, though definitive assignment cannot be made due to limited local resolution. The location and shape of this density are consistent with the LptM binding site identified in our structures.

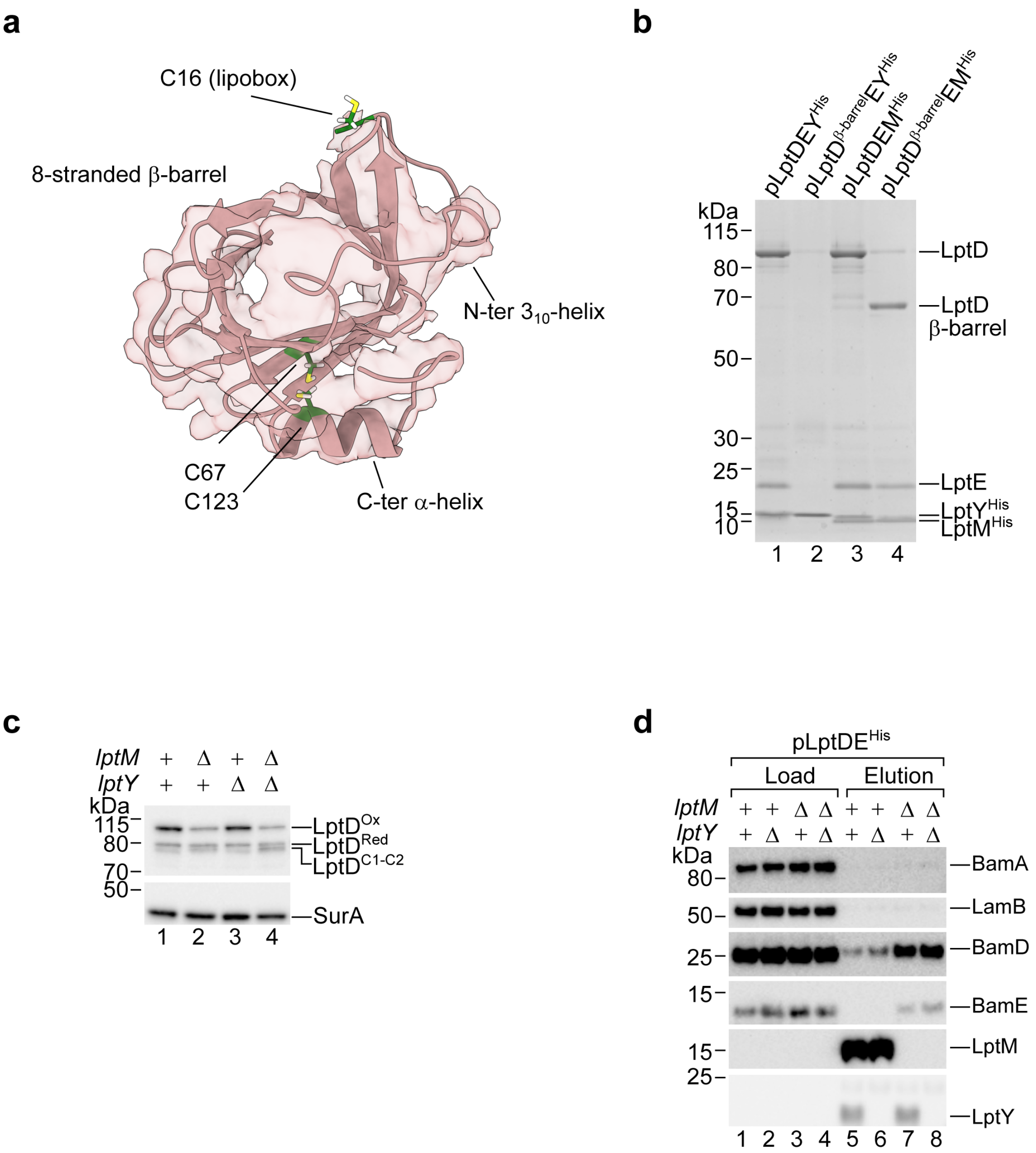

Supplementary Fig. 10. Characterization of LptY interaction with LptD

a, The AlphaFold 2-predicted model of LptY (shown in pink ribbon representation) is docked into its corresponding cryo-EM density map shown as a transparent surface. The main structural features of LptY are indicated. The lipobox cysteine (C16) that anchors LptY to the membrane, and the conserved cysteines (C67 and C123) that form an intramolecular disulfide bridge are highlighted as green sticks. **b**, Wild-type cells transformed with the indicated plasmids were subjected to envelope

fractionation, solubilization with DDM followed by nickel-affinity chromatography. Proteins in the elution fractions were separated by SDS-PAGE and stained with Coomassie Blue. The result is representative of three independent experimental repeats. **c**, Total cell lysates of the indicated strains were subjected to non-reducing SDS-PAGE and immunoblotting using the indicated antisera. The result is representative of three independent experimental repeats. **d**, The indicated strains transformed with pLptDE^{His} were subjected to envelope fractionation, solubilization with DDM followed by nickel-affinity purification of LptE^{His}. Load and elution fractions were subjected to SDS-PAGE and immunoblotting using the indicated antisera. Load: 0.1%; Elution 100%. The result is representative of three independent experimental repeats.

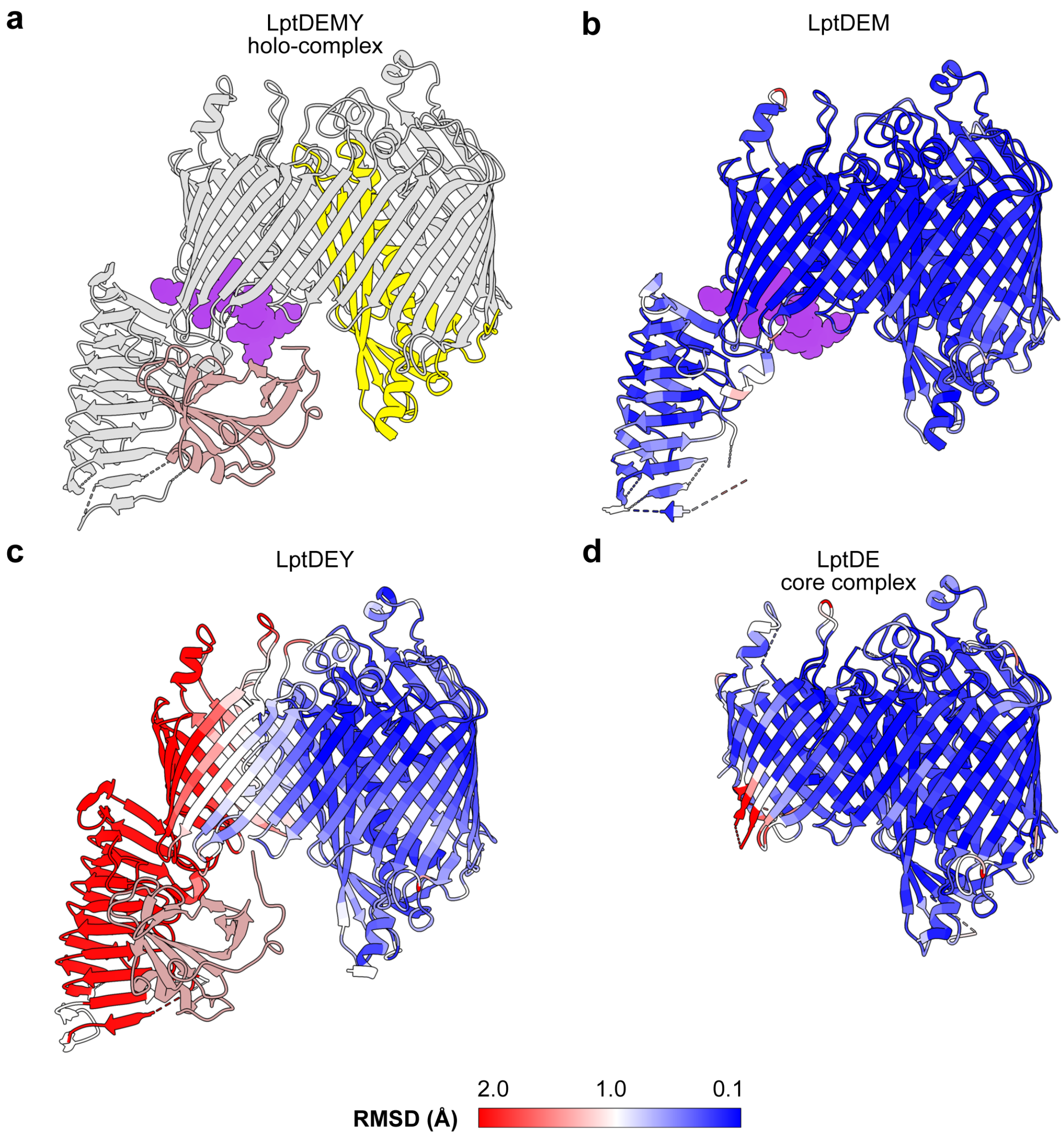

Supplementary Fig. 11. Structural comparison of translocon complexes

Root mean square deviation (RMSD) of the superimposed structures are shown for LptD and LptE: **a**, LptDEMY holo-complex is used as a common reference to calculate the RMSD values. **b**, LptDEM versus LptDEMY. **c**, LptDEY versus LptDEMY. **d**, LptDE core complex versus LptDEMY. b-d, RMSD data are shown on a blue to red scale and coloured onto a protein ribbon representation.

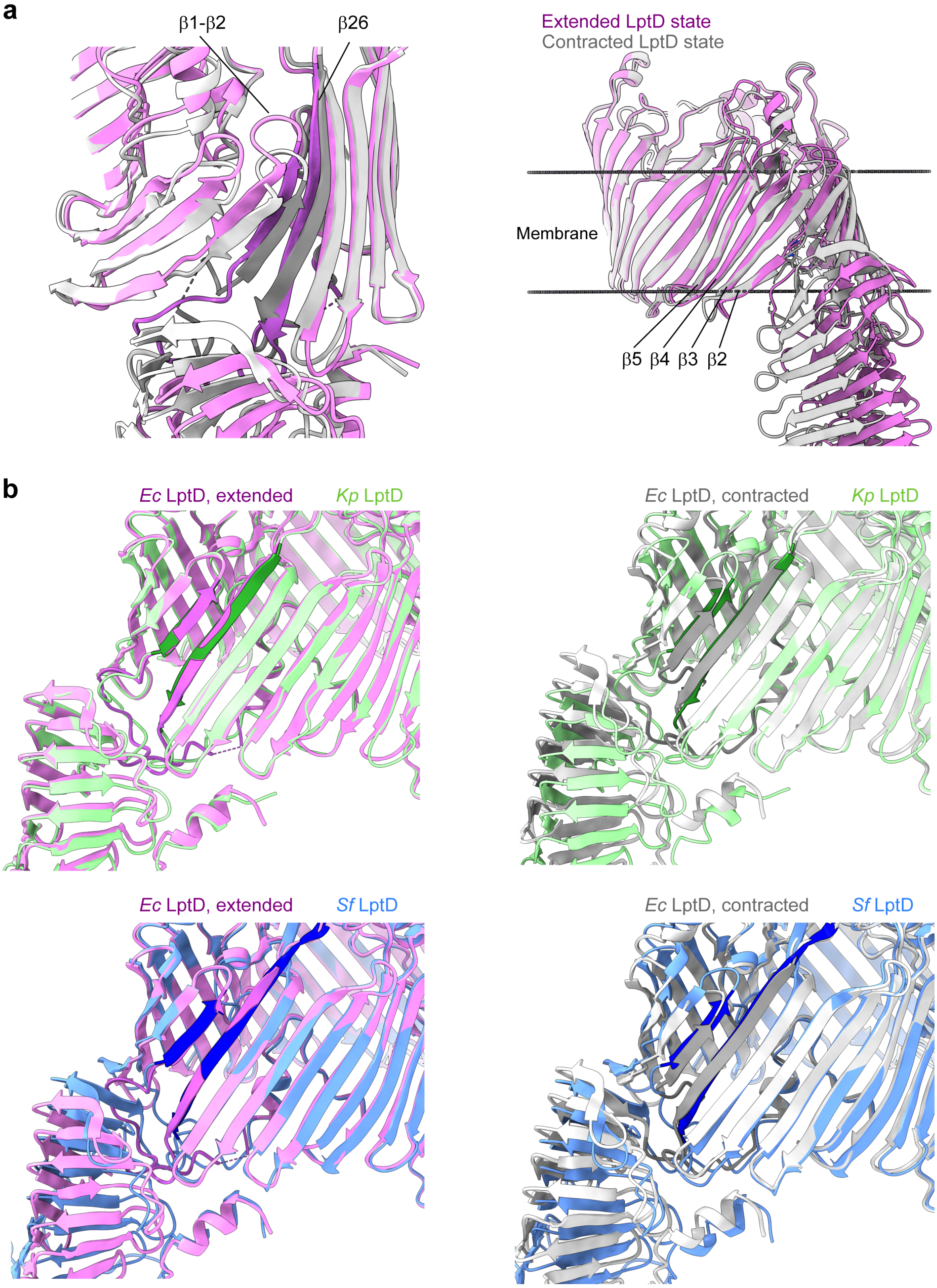
a
β1-β2
β26
Extended LptD state
Contracted LptD state
Membrane
β5 β4 β3 β2
b
Ec LptD, extended
Kp LptD
Ec LptD, contracted
Kp LptD
Ec LptD, extended
Sf LptD
Ec LptD, contracted
Sf LptD

Supplementary Fig. 12. Differential features of LptD states

a, Left, Superimposition of the structure of LptD extended (purple) and contracted (grey). β1 and β26 are shown in darker colours. **Right,** Superimposed LptD extended (purple) and contracted (grey) are predicted for membrane topology using the OPM database server [19]. The horizontal lines of grey dots represent the surfaces of a membrane lipid bilayer. **b**, Superimposition of the structure of LptD in the extended (purple) or contracted (grey) states with the structures of LptD from *Klebsiella pneumoniae* (green) or *Shigella flexneri* (blue). β1 and β26 are shown in darker colours.

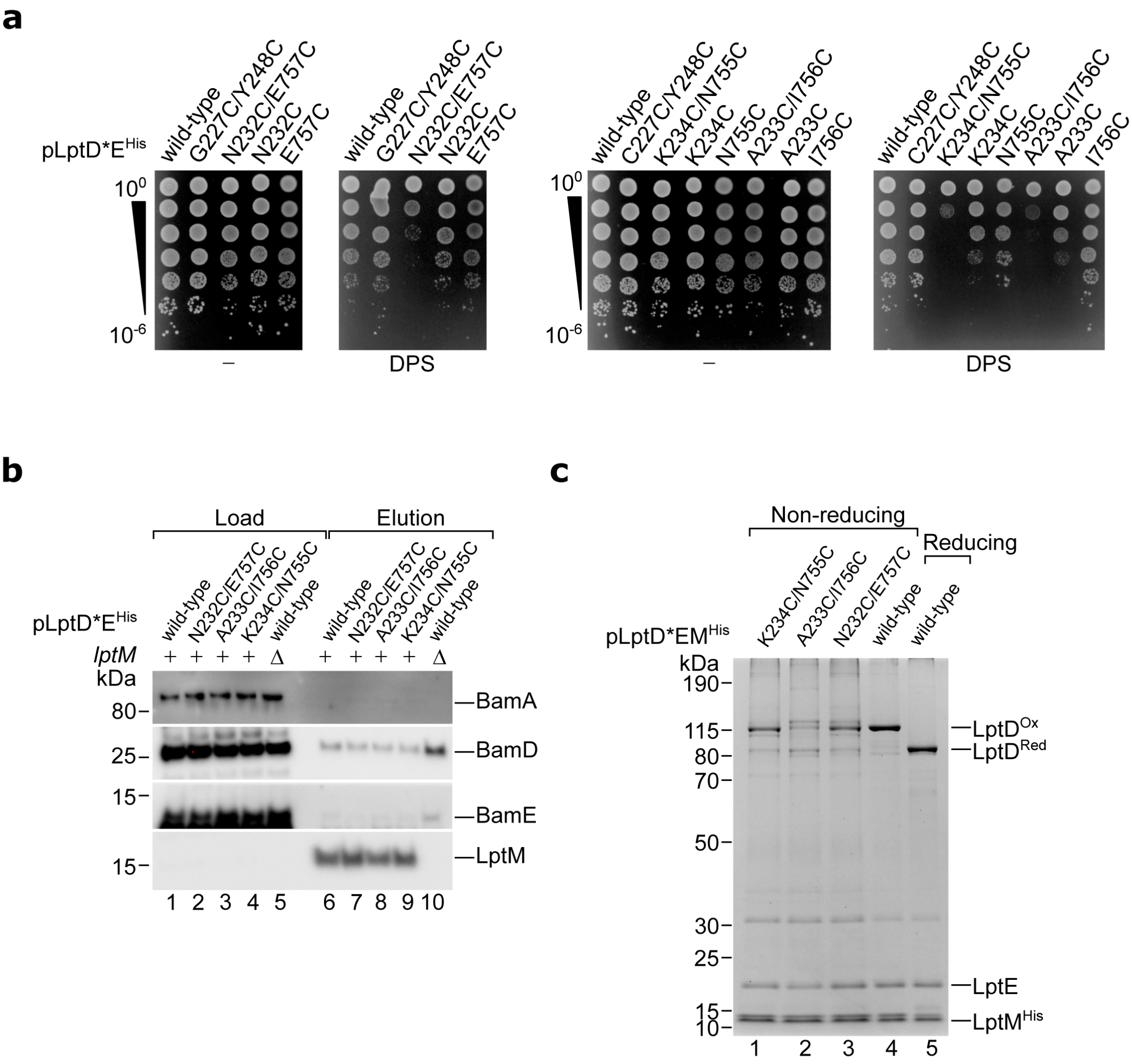

Supplementary Fig. 13. Analysis of LptD Cys mutations in β1 and β26

a, Cells deleted of chromosomal *lptD* and harbouring plasmid-borne wild-type *lptD* (pLptDE^{His}) or mutated *lptD* alleles encoding the indicated LptD variants (*) were progressively diluted and spotted on LB agar lacking (-) or supplemented with the oxidant 4-DPS. **b**, The strains tested in (a) were subjected to envelope fractionation, solubilization with DDM followed by nickel-affinity purification of LptE^{His}. Load and elution fractions were subjected to SDS-PAGE and immunoblotting using the indicated antisera. Load: 0.1%; Elution 100%. **c**, Cells deleted of chromosomal *lptD* and harbouring plasmid-borne wild-type LptD (pLptDEM^{His}) or mutated *lptD* alleles encoding the indicated LptD variants (*) were subjected to envelope fractionation, solubilization with DDM followed by nickel-affinity chromatography. Proteins in the elution fractions were treated with β-mercaptoethanol (reducing) or not treated (non

reducing), separated by SDS-PAGE and stained with Coomassie Blue. The results in all panels of this figure are representative of three independent experimental repeats.

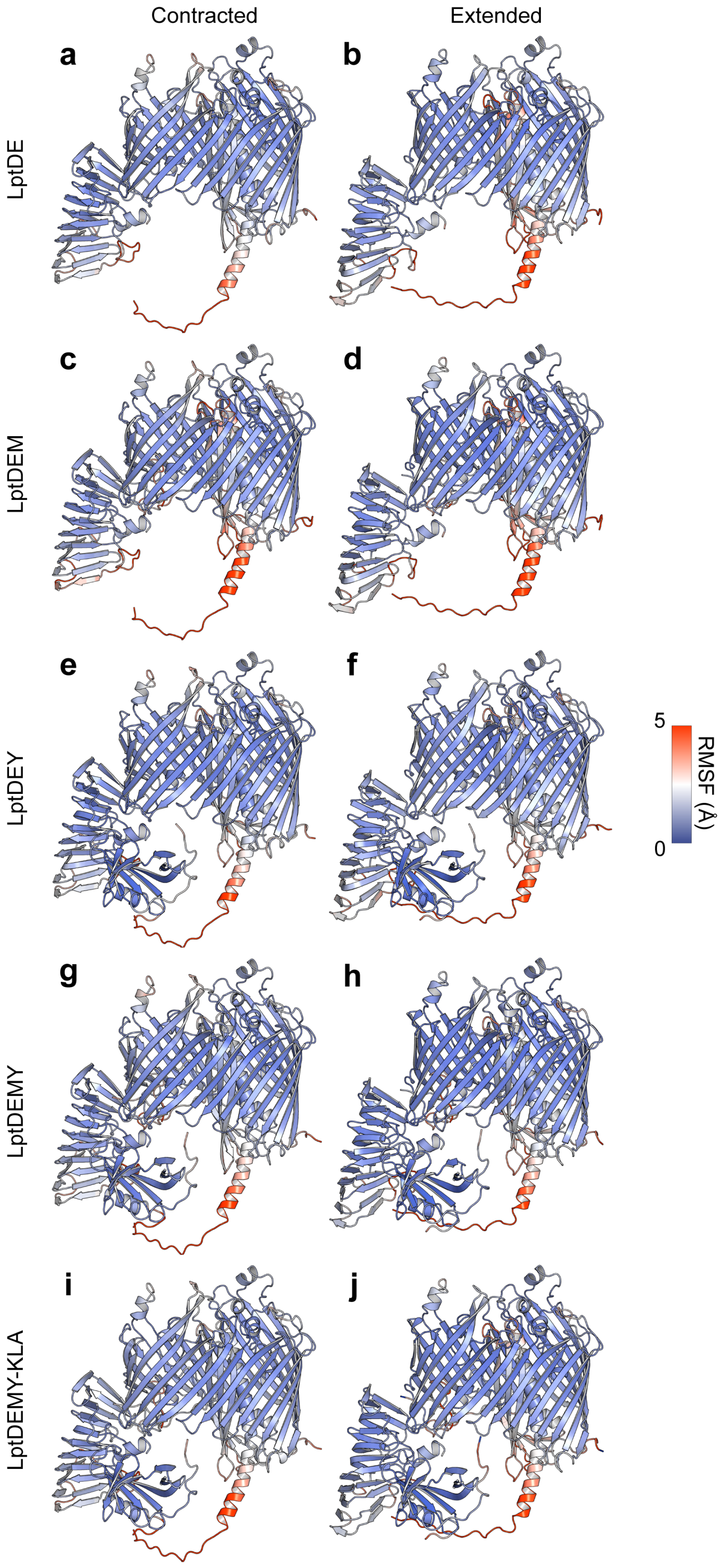

Contracted
Extended
a
b
c
d
e
f
g
h
i
j
LptDE
LptDEM
LptDEY
LptDEMY
LptDEMY-KLA
5
0
RMSF (Å)

Supplementary Fig. 14. RMSF of simulated complexes

Cα root mean square fluctuations (RMSF) of the protein complexes simulated in this study. Data is shown on a blue to red scale and coloured onto a protein ribbon representation. Simulations are shown for the five contracted (**a**, **c**, **e**, **g**, and **i**) and ex tended (**b**, **d**, **f, h**, and **j**) states for (a, b) LptDE, (c, d) LptDEM, (e, f,) LptDEY, (g, h) L ptDEMY, and (I, j) LptDEMY with bound KLA. Source data are available at the Zenodo repository, https://doi.org/10.5281/zenodo.16643358

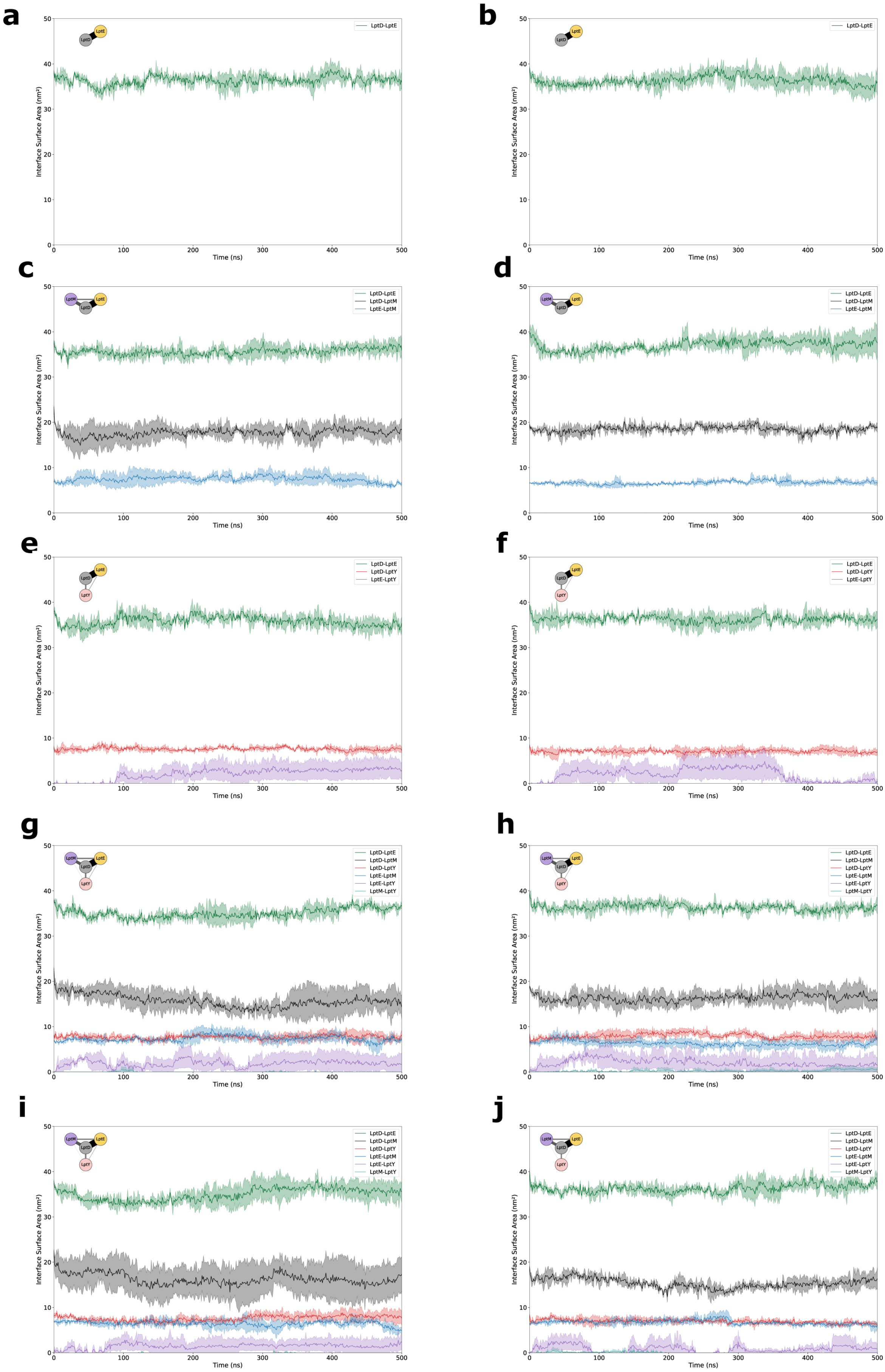
a
b
c
d
e
f
g
h
i
j
Interface Surface Area (nm²)
Time (ns)
LptD-LptE
LptD-LptM
LptD-LptY
LptE-LptM
LptE-LptY
LptM-LptY
LptD
LptE
LptM
LptY

Supplementary Fig. 15. Interface Solvent Accessible Surface Area between simulated complexes

The subunit interface was determined by calculating the combined solvent-accessible surface area (SASA) of the individual proteins and subtracting the SASA of the corresponding protein complex. The resulting value was then divided by two to estimate the surface area buried upon complex formation. This calculation was performed at each frame, to show the time-evolution of the SASA. Simulations are shown for the five contracted (**a**, **c**, **e**, **g**, and **i**) and extended (**b**, **d**, **f**, **h**, and **j**) states for (a, b) LptDE, (c, d) LptDEM, (e, f,) LptDEY, (g, h) LptDEMY, and (I, j) LptDEMY with bound KLA. The line graphs show the average SASA across the three independent repeats, with the standard deviation shown at each point. Thumbnail images at the top right of each panel illustrate the subunit composition of the complex. Interconnecting lines between subunits are displayed using a grey-to-black color scale, where increasing line thickness corresponds to greater interface SASA values, indicating more extensive surface burial upon complex formation. Source data are available at the Zenodo repository, https://doi.org/10.5281/zenodo.16643358

a

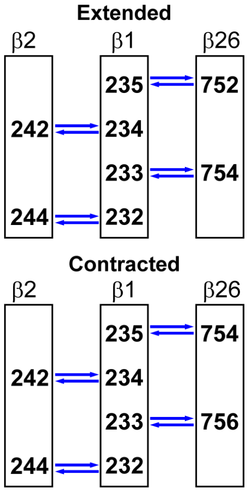

	Starting Conformation			
	Extended		Contracted	
	β2-β1	β1-β26	β2-β1	β1-β26
LptDE	5.04±0.70	3.31±0.68	3.85±1.0	3.62±0.65
LptDEM	4.81±0.79	2.63±0.83	2.92±0.84	1.98±0.70
LptDEY	4.90±0.69	3.38±0.74	3.99±0.99	3.29±0.63
LptDEMY	5.08±0.83	2.79±0.78	2.72±0.70	2.72±0.70
LptDEMY+KLA	5.17±0.81	2.01±0.46	2.36±0.52	2.36±0.52

b

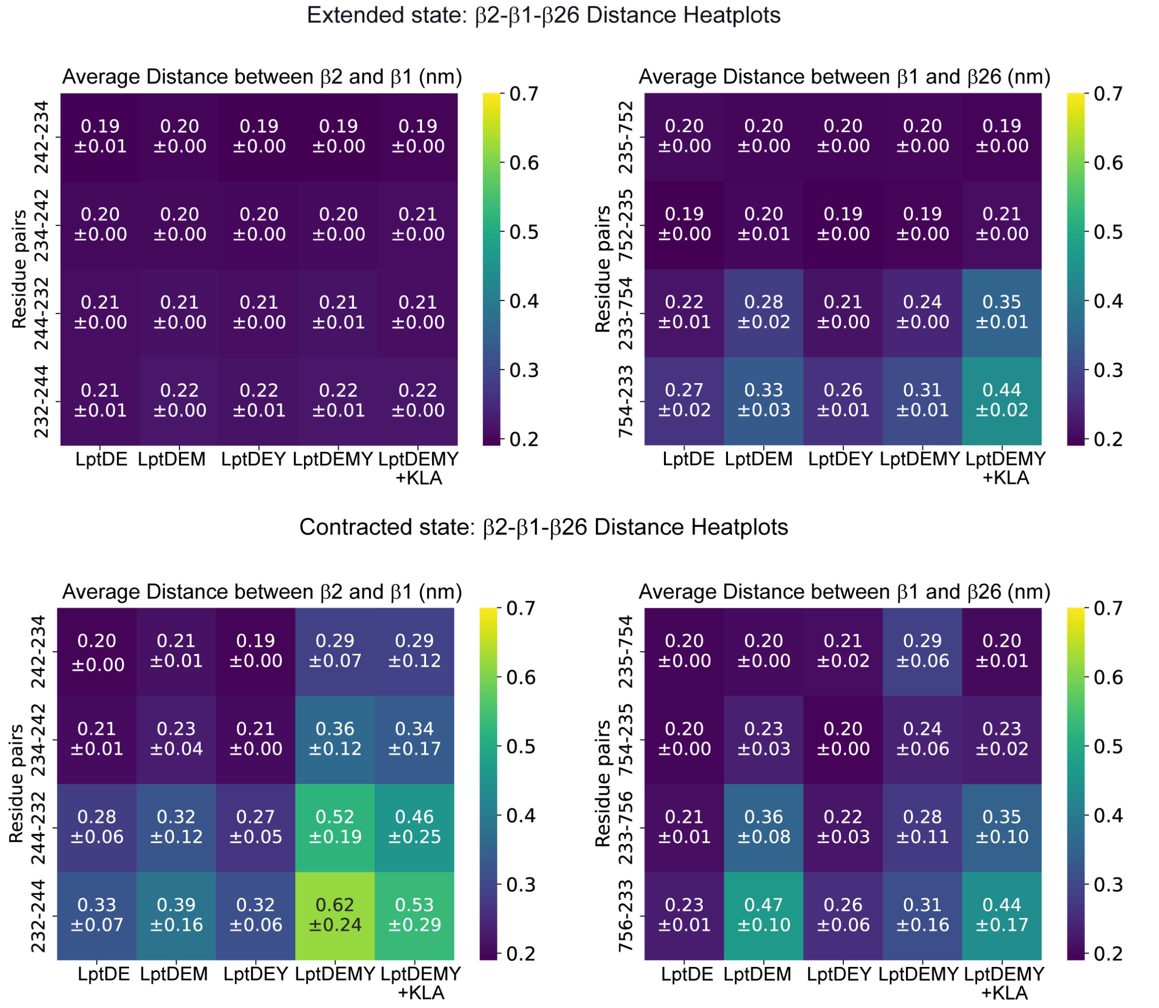

Supplementary Fig. 16. β2-β1-β26 pairing during MD simulations

a, **Left**, diagram illustrating β2-β1-β26 pairing in the extended and contracted LptD states. **Right**, average and standard deviation for the number of H-bonds computed during MD simulations for LptD in the extended state (LptDEM) or contracted state

(LptDEY) upon addition or removal of LptM or LptY or addition of KLA as indicated in each row (See also Supplementary Fig. 14). **b**, Average and standard deviations for the distances between β2-β1 or β1-β26 were calculated for the indicated complexes during MD simulations described in a). For each residue pair, two hydrogen bond distances were calculated: from the C=O of one residue to the N–H of the other, and vice versa. Source data are available at the Zenodo repository, https://doi.org/10.5281/zenodo.16643358

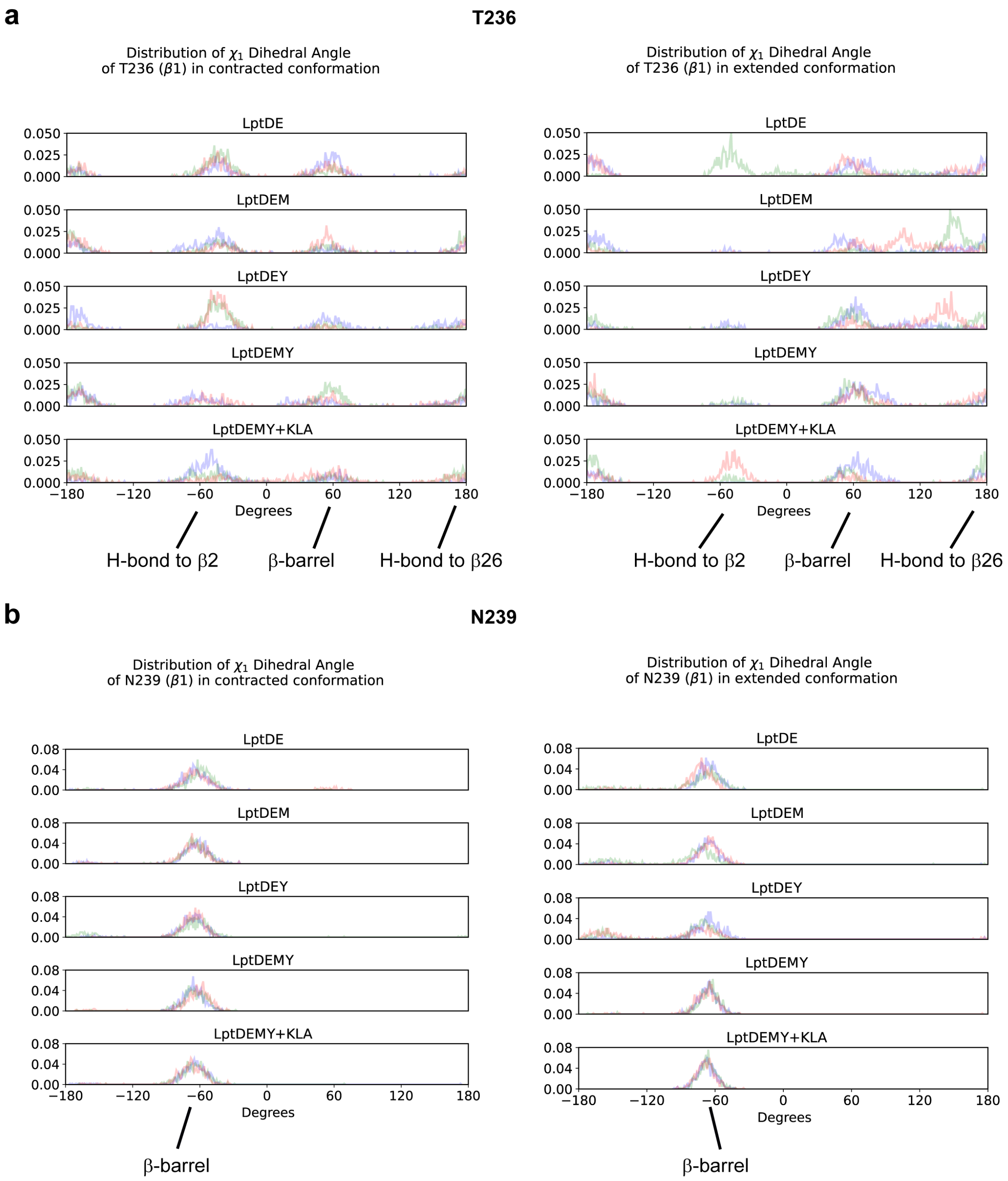

Supplementary Fig. 17. T236 and N239 dihedral angle distributions

Distribution of dihedral angle of LptD T236 (**a**) and LptD N239 (**b**) during three independent MD simulations runs. Source Data are available at the Zenodo repository, https://doi.org/10.5281/zenodo.16643358

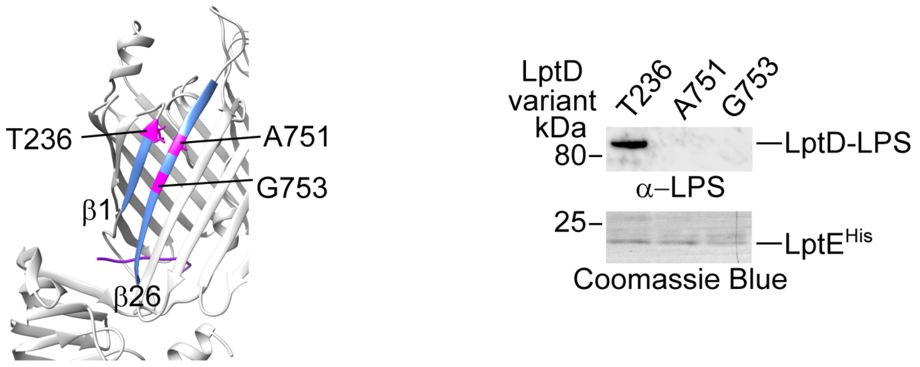

Supplementary Fig. 18. Preferential LPS-interaction site at the lateral gate N-terminal side

Left, The structure of LptDEM (extended state) is shown in ribbon representation to highlight (purple) the LptD amino acids replaced with pBpa for site-directed photocrosslinking; **Right**, UV induced photocrosslinking of the indicated strains transformed with pLptDE^{His} harbouring amber mutations at the indicated amino acid positions of LptD. Upon in vivo photocrosslinking, the envelope fractions were solubilized with DDM and subjected to nickel-affinity purification of LptE^{His}. The result is representative of three independent experimental repeats.

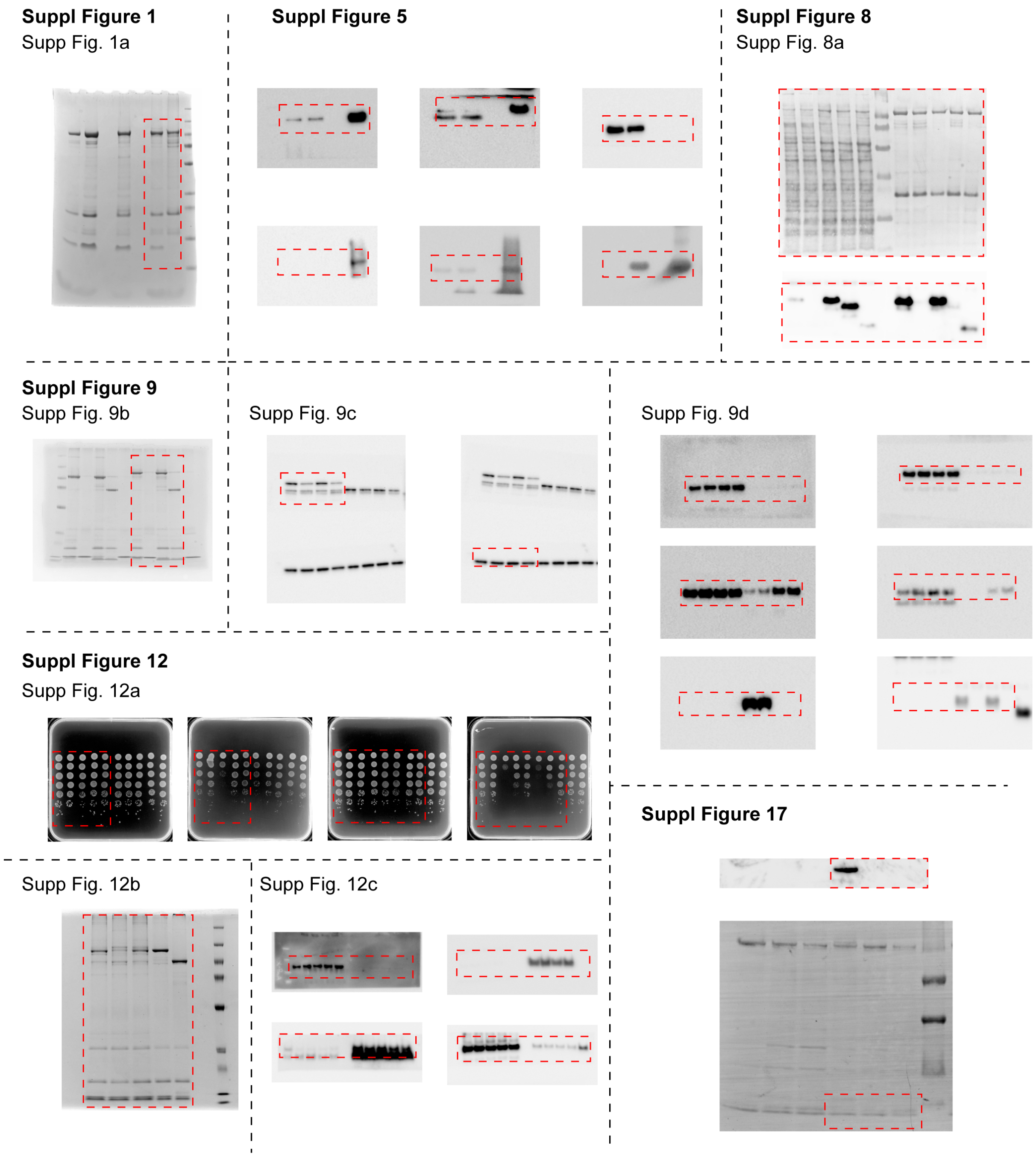

Supplementary Fig. 19. Uncropped gels, membranes and Drop tests of Supplementary Figures.

Red boxes indicate areas cropped and presented in the supplementary figures as indicated. For gels, the staining method and antibodies are reported in the corresponding Figure Legends

SUPPLEMENTARY TABLES

Supplementary Table 1. LptD – LptM contacts

The interface contacts between LptD and LptM were determined in ChimeraX by identifying the pairs of atoms between the experimental model of LptD and the AlphaFold 2 in silico model of LptY with a Van der Waals overlap > -0.4 A.

LptDEM

LptM #	LptM AA	LptD #	LptD AA	contacts
20	CYS	216	LEU	1
20	CYS	217	GLN	4
20	CYS	219	PRO	1
20	CYS	228	PHE	3
21	GLY	219	PRO	1
21	GLY	225	ARG	4
21	GLY	227	GLY	1
21	GLY	228	PHE	3
22	LEU	219	PRO	5
22	LEU	221	GLY	1
22	LEU	223	LYS	2
22	LEU	224	ARG	2
22	LEU	225	ARG	6
22	LEU	248	TYR	6
23	LYS	248	TYR	8
23	LYS	258	THR	2
23	LYS	275	GLU	4
24	GLY	224	ARG	5
24	GLY	277	ARG	3
25	PRO	224	ARG	3
26	LEU	253	PRO	1
26	LEU	254	ASN	2
26	LEU	255	MET	3
26	LEU	256	ASP	3
26	LEU	277	ARG	4
26	LEU	278	TYR	3
27	TYR	253	PRO	10
27	TYR	254	ASN	4
27	TYR	279	LEU	3
28	PHE	279	LEU	7
28	PHE	280	SER	2
29	PRO	253	PRO	2
29	PRO	254	ASN	2
			total	107

LptDEMY

LptM #	LptM AA	LptD #	LptD AA	contacts
20	CYS	217	GLN	3
20	CYS	228	PHE	3
21	GLY	219	PRO	1
21	GLY	225	ARG	4
21	GLY	227	GLY	1
21	GLY	228	PHE	3
21	GLY	229	LEU	4
22	LEU	219	PRO	4
22	LEU	221	GLY	1
22	LEU	223	LYS	1
22	LEU	224	ARG	4
22	LEU	225	ARG	4
22	LEU	248	TYR	3
23	LYS	224	ARG	1
23	LYS	248	TYR	9
23	LYS	258	THR	1
23	LYS	275	GLU	4
24	GLY	224	ARG	5
24	GLY	277	ARG	3
25	PRO	224	ARG	4
26	LEU	224	ARG	4
26	LEU	253	PRO	2
26	LEU	254	ASN	2
26	LEU	255	MET	1
26	LEU	256	ASP	1
26	LEU	277	ARG	2
26	LEU	278	TYR	4
26	LEU	279	LEU	1
27	TYR	224	ARG	3
27	TYR	253	PRO	10
27	TYR	254	ASN	3
28	PHE	279	LEU	5
28	PHE	280	SER	2
29	PRO	253	PRO	3
29	PRO	254	ASN	1
			total	107

Supplementary Table 2. LptD – LptY contacts

The interface contacts between LptD and LptY were determined in ChimeraX by identifying the pairs of atoms between the experimental model of LptD and the AlphaFold 2 in silico model of LptY with a Van der Waals overlap > -0.4 A.

LptDEY

LptY #	LptY AA	LptD #	LptD AA	contacts
16	CYS	646	ARG	27
17	ALA	646	ARG	1
18	GLU	619	ARG	14
18	GLU	646	ARG	3
48	VAL	30	GLN	4
48	VAL	174	LEU	4
68	ARG	33	LEU	8
69	GLN	33	LEU	1
70	TRP	33	LEU	4
70	TRP	34	GLY	8
70	TRP	174	LEU	6
70	TRP	175	PRO	19
71	GLN	33	LEU	2
71	GLN	34	GLY	13
71	GLN	36	PRO	1
71	GLN	175	PRO	4
72	ARG	32	MET	18
72	ARG	33	LEU	1
72	ARG	35	VAL	4
72	ARG	37	SER	5
74	ILE	29	SER	2
74	ILE	33	LEU	4
95	GLU	25	ALA	3
97	TYR	25	ALA	4
			total	133

LptDEMY

LptY #	LptY AA	LptD #	LptD AA	contacts
18	GLU	619	ARG	9
18	GLU	646	ARG	11
48	VAL	30	GLN	2
48	VAL	33	LEU	1
48	VAL	174	LEU	3
68	ARG	33	LEU	8
69	GLN	33	LEU	2
70	TRP	33	LEU	2
70	TRP	34	GLY	3
70	TRP	174	LEU	3
70	TRP	175	PRO	8
71	GLN	33	LEU	1
71	GLN	34	GLY	6
71	GLN	36	PRO	1
71	GLN	141	GLN	3
71	GLN	175	PRO	4
72	ARG	32	MET	5
72	ARG	35	VAL	6
72	ARG	36	PRO	1
72	ARG	37	SER	3
74	ILE	29	SER	1
74	ILE	32	MET	1
74	ILE	33	LEU	6
97	TYR	25	ALA	4
109	ASP	25	ALA	6
			total	94

Supplementary Table 3. List of bacterial strains

**Strain name**	**Reference**	**Lab Identifier**
BW25113: Δ(*araD-araB*)*567* Δ(*rhaD-rhaB*)*568 ΔlacZ4787*(::rrnB-3) *hsdR514 rph-1 (wild-type reference)*	[20]	Y1
BW25113 *lptM::kan*	[10]	Y14
BW25113 Δ*lptM*	[21]	Y40
BW25113 *lptY::kan*	This study	Y-CHX51
BW25113 Δ*lptY*	This study	Y-CHX15
BW25113 *ΔlptM lptY::kan*	This study	Y-CHX49
BW25113 *dsbA::kan*	[10]	Y81
BW25113 *ΔlptY dsbA::kan*	This study	Y-CHX52
BW25113 *lptD::kan ;* pLptDE^{His}	This study	Y-P337
BW25113 *lptD::kan ;* pLptD$^{G227C-Y248C}$ E^{His}	This study	Y-P349
BW25113 *lptD::kan ;* pLptD$^{N232C-E757C}$ E^{His}	This study	Vio-369
BW25113 *lptD::kan ;* pLptD^{N232C} E^{His}	This study	Vio-372
BW25113 *lptD::kan ;* pLptD^{E757C} E^{His}	This study	Vio-375
BW25113 *lptD::kan ;* pLptD$^{A233C-I756C}$ E^{His}	This study	Y-P352
BW25113 *lptD::kan ;* pLptD^{A233C} E^{His}	This study	Vio-360
BW25113 *lptD::kan ;* pLptD^{I756C} E^{His}	This study	Vio-363
BW25113 *lptD::kan ;* pLptD$^{K234C-N755C}$ E^{His}	This study	Y-P346
BW25113 *lptD::kan ;* pLptD^{K234C} E^{His}	This study	Vio-346
BW25113 *lptD::kan ;* pLptD^{N755C} E^{His}	This study	Vio-349
BW25113 *lptD::kan ;* pLptDEM^{His}	This study	Vio-388
BW25113 *lptD::kan ;* pLptD$^{N232C-E757C}$ EM^{His}	This study	Vio-391
BW25113 *lptD::kan ;* pLptD$^{A233C-I756C}$ EM^{His}	This study	Vio-390
BW25113 *lptD::kan ;* pLptD$^{K234C-N755C}$ EM^{His}	This study	Vio-392
BW25113 *ΔlptM lptD::kan ;* pLptDE^{His}	This study	Y-P359

Supplementary Table 4. List of Plasmids

Plasmid name	Cloning strategies used in this study	Reference	Lab Identifier
pCtrl (empty vector)		[21]	pV3
pLptDME^{His}		[10]	pYY40
pLptDM$^{\Delta 22\text{-}30}$E^{His}	Recombinational cloning	This study	pVM113
pLptDM$^{\Delta 44\text{-}67}$E^{His}	Inverse PCR	This study	pVM90
pLptDE^{His}		[10]	pYY68
pLptD$^{G227C\text{-}Y248C}$ E^{His}	Site-directed mutagenesis	This study	pVM132
pLptD$^{N232C\text{-}E757C}$ E^{His}	Recombinational cloning	This study	pVM204
pLptD^{N232C} E^{His}	Site-directed mutagenesis	This study	pVM197
pLptD^{E757C} E^{His}	Site-directed mutagenesis	This study	pVM203
pLptD$^{A233C\text{-}I756C}$ E^{His}	Site-directed mutagenesis	This study	pVM135
pLptD^{A233C} E^{His}	Recombinational cloning	This study	pVM198
pLptD^{I756C} E^{His}	Recombinational cloning	This study	pVM199
pLptD$^{K234C\text{-}N755C}$ E^{His}	Site-directed mutagenesis	This study	pVM186
pLptD^{K234C} E^{His}	Recombinational cloning	This study	pVM195
pLptD^{N755C} E^{His}	Recombinational cloning	This study	pVM196
pLptD^{Y112am} E^{His}	Site-directed mutagenesis	This study	pVM123
pLptD^{F203am} E^{His}	Recombinational cloning	This study	pVM174
pLptD^{V220am} E^{His}	Site-directed mutagenesis	This study	pVM124
pLptD^{F228am} E^{His}	Site-directed mutagenesis	This study	pVM125
pLptD^{I230am} E^{His}	Recombinational cloning	This study	pVM175
pLptD^{N232am} E^{His}	Recombinational cloning	This study	pVM176
pLptD^{T236am} E^{His}	Site-directed mutagenesis	This study	pVM126
pLptD^{N239am} E^{His}	Site-directed mutagenesis	This study	pCHX33
pLptD^{A751am} E^{His}	Site-directed mutagenesis	This study	pVM189
pLptD^{G753am} E^{His}	Site-directed mutagenesis	This study	pVM190
pLptDEM^{His}		[10]	pYY43
pLptD$^{N232C\text{-}E757C}$ EM^{His}	Recombinational cloning	This study	pVM208
pLptD$^{A233C\text{-}I756C}$ EM^{His}	Recombinational cloning	This study	pVM206
pLptD$^{K234C\text{-}N755C}$ EM^{His}	Recombinational cloning	This study	pVM210
pLptD^{Y112am} EM^{His}	Site-directed mutagenesis	This study	pHX10
pLptD^{F228am} EM^{His}	Site-directed mutagenesis	This study	pHX11
pLptD^{T236am} EM^{His}	Site-directed mutagenesis	This study	pHX12
pLptD$^{\beta\text{-barrel}}$EM^{His}		[10]	pVM52
pLptD$^{\beta\text{-barrel}}$E-LptY^{His}	Recombinational cloning	This study	pCHX09
pLptDE-LptY^{His}	Recombinational cloning	This study	pCHX02
pLptY^{His}	Recombinational cloning	This study	pCHX01
pLptC^{His}		[10]	pVM83
pEVOL-pBpF		[22]	pEVOL-pBpF
pKOBEG		[23]	pKOBEG

Supplementary Table 5. List of oligonucleotides

Oligonucleotide name	Sequence	Lab Identifier
LptM$^{\Delta 22-30}$-Fw	GCAGATAAAAACGCACCGCCG	Ovio104
LptM$^{\Delta 22-30}$-Rev	TGCGTTTTTATCTGCACCGCAGCCCGTCAGGC	Ovio103
LptM$^{\Delta 44-67}$-Fw	TAAGCAGCGGCCGGCG	LptM-44-67
LptM$^{\Delta 44-67}$-Rev	CTCTACCGGTTTGGTCGGCG	Ovio60
LptD-K234C-Fw	ATCCCGAACGCCTGCTACACCACCACCAACTAC	Ovio132
LptD-K234C-Rev	GGCGTTCGGGATCAAGAAACC	Ovio214
LptD-N755C-Fw	CGCAATCGGCTTTTGCATCGAACTTCGCG	Ohx78
LptD-N755C-Rev	CGCGAAGTTCGATGCAAAAGCCGATTGCG	Ohx79
LptD-A233C-Fw	TCTTGATCCCGAACTGCAAGTACACCACCACC	Ovio130
LptD-A233C-Rev	TGGTGGTGTACTTGCAGTTCGGGATCAAGAAACC	Ovio129
LptD-I756C-Fw	ATCGGCTTTAACTGCGAACTTCGCGGCC	Ovio139
LptD-I756C-Rev	CGCGAAGTTCGCAGTTAAAGCCGATTGC	Ovio140
LptD-N232C-Fw	CTTGATCCCGTGCGCCAAGTACACCACC	Ohx76
LptD-N232C-Rev	GGTGGTGTACTTGGCGCACGGGATCAAG	Ohx77
LptD-E757C-Fw	ATCGGCTTTAACATCTGCCTTCGCGGCCTGAG	Ovio236
LptD-E757C-Rev	GATGTTAAAGCCGATTGCGTTGTC	Ovio237
LptD-G227C-Fw	ACAAACGTCGCTCTTGTTTCTTGATCCC	Ovio125
LptD-G227C-Rev	ATCAAGAAACAAGAGCGACGTTTGTCAC	Ovio126
LptD-Y248C-Fw	ACCTGCCATATTGCTGGAACATCGC	Ovio138
LptD-Y248C-Rev	CGCGATGTTCCAGCAATATGGCAGGTAG	Ovio137
LptD-Y112am-Fw	CGGTAATGTCCATTAGGACGATAACCAGG	Ovio114
LptD-Y112am-Rev	ACCTGGTTATCGTCCTAATGGACATTACCGAGC	Ovio115
LptD-F203am-Fw	ATCTGGAACGCCCGCTAGAAGGTGGGTC	Ovio215
LptD-F203am-Rev	GCGGGCGTTCCAGATCTCC	Ovio216
LptD-V220am-Fw	TTGCAGTTGCCGTAGGGTGACAAACGTC	Ovio116
LptD-V220am-Rev	CGACGTTTGTCACCCTACGGCAACTGC	Ovio117
LptD-F228am-Fw	ACGTCGCTCTGGTTAGTTGATCCCGAACG	Ovio118
LptD-F228am-Rev	GCGTTCGGGATCAACTAACCAGAGCGACG	Ovio119
LptD-I230am-Fw	TCGCTCTGGTTTCTTGTAGCCGAACGCCAAGTAC	Ovio209
LptD-I230am-Rev	AAGAAACCAGAGCGACGTTTGTC	Ovio210
LptD-N232am-Fw	TGGTTTCTTGATCCCGTAGGCCAAGTACACCAC	Ovio211
LptD-N232am-Rev	GGGATCAAGAAACCAGAGCGAC	Ovio212
LptD-T236am-Fw	CCCGAACGCCAAGTACTAGACCACCAACTACTT	Ovio120
LptD-T236am-Rev	GTAGTTGGTGGTCTAGTACTTGGCGTTCG	Ovio121

	GGATC	
LptD-N239am-Fw	TACACCACCACCTAGTACTTTGAGTTCTACCTG	Ovio160
LptD-N239am-Rev	CTAGGTGGTGGTGTACTTGGCG	Ovio161
LptD-A751am-Fw	GCGGTATATGACAACTAGATCGGCTTTAACATCGAAC	Ovio233
LptD-A751am-Rev	GTTGTCATATACCGCATGTTGTTTAT	Ovio231
LptD-G753am-Fw	GCGGTATATGACAACGCAATCTAGTTTAACATCGAACTTC	Ovio234
LptD-G753am-Rev	GTTGTCATATACCGCATGTTGTTTAT	Ovio231
LptD-Nter-RC-Fw	CCCCACTCTCCTGGCCAC	Ovio152
LptD-Nter-RC-Rev	GCCAGGAGAGTGGGGATACG	Ovio153
LptD-Cter-RC-Fw	TGCGTTCGAACATTCTGCCG	Ovio154
LptD-Cter-RC-Rev	GAATGTTCGAACGCAGCATCTC	Ovio155
LptD-Int-RC-Fw	CCAAATATGGATGCCACCATCACG	Ovio232
LptD-Int-RC-Rev	GGCATCCATATTTGGCGCG	Ovio204
LptY-Fw	GAACGCATAATAACGATGAAAAAACTCGCAATTGCAGGC	Ohx3
LptY-Rev	ATGGTGGTGATGATGCGGCAGTGGCGTCGGTAA	Ohx4
LptD-kanR-Fw (λ red recombination)	ACCGTTTGTCACGCGCAACGTTACCGATGATGGAACAATAAGTGTAGGCTGGAGCTGC	YAA209
LptD-kanR-Rev (λ red recombination)	TAACCGCACTGCGGATTACGTGGTAAATCAACAAATCACACATATGAATATCCTCCTTAGTTCCTATTCC	YAA210

Supplementary Table 6. CryoEM data collection, processing and modelling statistics

pa	LptDE barrel	LptDEY	LptDEM	LptDEMY
PDB	9I9Z	9IA0	9IA2	9IA5
EMDB	52773	52777	52778	52779
**Data collection**				
Microscope	Titan Kryos			
Camera	Falcon 4i			
Voltage (kV)	300			
magnification	x165000			
total dose (e-/Å2)	50			
Pixel size (Å)	0.731			
Defocus range (µm)	-0.5 to -2.5			
**Processing**				
Symmetry imposed	C1	C1	C1	C1
Micrographs number	20832		18816	
Initial particle images (no.)	5,219,710	6,375,528	909,602	302,650
Final particle images (no.)	107,410	127,785	198,109	146,385
Map resolution (Å) – (0.143 FSC threshold model)	2.74	2.62	2.47	2.63
**Refinement and validation**				
Map sharpening (B-factor) ($Å^{-2}$)	45.7	93.2	61.7	62.1
**Model Composition**				
No. of chains	2	2	3	3
Atoms (no.)	10807	14071	13858	14067
Aminoacid Residues (no.)	680	894	877	891
Bond lengths (Å)	0.013	0.012	0.013	0.012
Bond angles (°)	1.799	1.791	1.782	1.844
Ramachandran outliers (%)	0	0.45	0	0.11

Ramachandran allowed (%)	2.85	2.49	2.33	2.63
Ramachandran favored (%)	97.15	97.06	97.67	97.26
Rotamer outliers (%)	1.51	1.02	0.52	1.02
MolProbity score	0.86	0.8	0.6	0.7
Clash score	0.19	0.36	0.07	0.14
CC (mask)	0.73	0.75	0.83	0.83
CC (box)	0.5	0.46	0.63	0.63
CC (peaks)	0.43	0.42	0.55	0.55
CC (volume)	0.73	0.73	0.82	0.82
Mean CC for ligands	---	---	---	---

Supplementary Table 7. MD simulations checklist

Information regarding reproducibility of the MD simulations.

**Reliability and reproducibility checklist for molecular dynamics simulations** ***All boxes must be marked YES by acceptance unless an N/A option is available**	**Yes**	**N/A**	**Response (Please state where this information can be found in the text)**
**1. Convergence of simulations and analysis**			
1a. Is an evaluation presented in the text to show that the property being measured has equilibrated in the simulations (*e.g.* time-course analysis)?	☒		Methods / SI Fig 14
1b. Then, is it described in the text how simulations are split into equilibration and production runs and how much data were analyzed from production runs?	☒		Methods
1c. Are there at least 3 simulations per simulation condition with statistical analysis?	☒		Methods
1d. Is evidence provided in the text that the simulation results presented are independent of initial configuration?	☒		SI Fig 14
**2. Connection to experiments**			
2a. Are calculations provided that can connect to experiments (*e.g.* loss or gain in function from mutagenesis, binding assays, NMR chemical shifts, J-couplings, SAXS curves, interaction distances or FRET distances, structure factors, diffusion coefficients, bulk modulus and other mechanical properties, *etc.*)?	☒		Methods
**3. Method choice**			
3a. Is it described in the text what force field and water model are used and why?	☒		Methods
3b. Do simulations contain membranes, membrane proteins, intrinsically disordered proteins, glycans, nucleic acids, polymers, or cryptic ligand binding?	☒	☐	Response not needed if **N/A**
If 3b is **YES**, are enhanced sampling methods used?	☐	☒	Response not needed if **N/A**
If enhanced sampling methods are used, are the convergence criteria clearly stated?	☐		
If 3b is **YES**, is it explained in the text why or why not enhanced sampling methods are used?	☒		Not appropriate
**4. Code and reproducibility**			
4a. Is a table provided describing the system setup, such as simulation box dimensions, total number of atoms, total number of water molecules, salt concentration, lipid composition (number of molecules and type)?	☒		Link to Zenodo
4b. Is it described in the text what simulation and analysis software and which versions are used?	☒		Methods
4c. Are initial coordinate and simulation input files and a coordinate file of the final output provided as	☒		Link to Zenodo

supplementary files or in a public repository?			
4d. Is there custom code or custom force field parameters?	☐	☒	Response not needed if **N/A**
If **YES**, are they provided as supplementary profiles or in a public repository?	☐		

SUPPLEMENTARY REFERENCES

1 Parks, D. H., Chuvochina, M., Rinke, C. *et al.* GTDB: an ongoing census of bacterial and archaeal diversity through a phylogenetically consistent, rank normalized and complete genome-based taxonomy. *Nucleic acids research* **50**, D785-d794, doi:10.1093/nar/gkab776 (2022).

2 Seemann, T. Prokka: rapid prokaryotic genome annotation. *Bioinformatics (Oxford, England)* **30**, 2068-2069, doi:10.1093/bioinformatics/btu153 (2014).

3 Emms, D. M. & Kelly, S. OrthoFinder: phylogenetic orthology inference for comparative genomics. *Genome biology* **20**, 238, doi:10.1186/s13059-019-1832-y (2019).

4 Katoh, K. & Standley, D. M. MAFFT multiple sequence alignment software version 7: improvements in performance and usability. *Molecular biology and evolution* **30**, 772-780, doi:10.1093/molbev/mst010 (2013).

5 Minh, B. Q., Schmidt, H. A., Chernomor, O. *et al.* IQ-TREE 2: New Models and Efficient Methods for Phylogenetic Inference in the Genomic Era. *Molecular biology and evolution* **37**, 1530-1534, doi:10.1093/molbev/msaa015 (2020).

6 Letunic, I. & Bork, P. Interactive Tree Of Life (iTOL) v5: an online tool for phylogenetic tree display and annotation. *Nucleic acids research* **49**, W293-w296, doi:10.1093/nar/gkab301 (2021).

7 Adeolu, M., Alnajar, S., Naushad, S. *et al.* Genome-based phylogeny and taxonomy of the 'Enterobacteriales': proposal for Enterobacterales ord. nov. divided into the families Enterobacteriaceae, Erwiniaceae fam. nov., Pectobacteriaceae fam. nov., Yersiniaceae fam. nov., Hafniaceae fam. nov., Morganellaceae fam. nov., and Budviciaceae fam. nov. *International journal of systematic and evolutionary microbiology* **66**, 5575-5599, doi:10.1099/ijsem.0.001485 (2016).

8 Parte, A. C., Sardà Carbasse, J., Meier-Kolthoff, J. P. *et al.* List of Prokaryotic names with Standing in Nomenclature (LPSN) moves to the DSMZ. *International journal of systematic and evolutionary microbiology* **70**, 5607-5612, doi:10.1099/ijsem.0.004332 (2020).

9 Finn, R. D., Clements, J. & Eddy, S. R. HMMER web server: interactive sequence similarity searching. *Nucleic acids research* **39**, W29-37, doi:10.1093/nar/gkr367 (2011).

10 Yang, Y., Chen, H., Corey, R. A. *et al.* LptM promotes oxidative maturation of the lipopolysaccharide translocon by substrate binding mimicry. *Nature communications* **14**, 6368, doi:10.1038/s41467-023-42007-w (2023).

11 Capella-Gutiérrez, S., Silla-Martínez, J. M. & Gabaldón, T. trimAl: a tool for automated alignment trimming in large-scale phylogenetic analyses. *Bioinformatics (Oxford, England)* **25**, 1972-1973, doi:10.1093/bioinformatics/btp348 (2009).

12 Kalyaanamoorthy, S., Minh, B. Q., Wong, T. K. F. *et al.* ModelFinder: fast model selection for accurate phylogenetic estimates. *Nature methods* **14**, 587-589, doi:10.1038/nmeth.4285 (2017).

13 Wilm, M., Shevchenko, A., Houthaeve, T. *et al.* Femtomole sequencing of proteins from polyacrylamide gels by nano-electrospray mass spectrometry. *Nature* **379**, 466-469, doi:10.1038/379466a0 (1996).

14 Bouyssié, D., Dubois, M., Nasso, S. *et al.* mzDB: a file format using multiple indexing strategies for the efficient analysis of large LC-MS/MS and SWATH-MS data sets. *Molecular & cellular proteomics : MCP* **14**, 771-781, doi:10.1074/mcp.O114.039115 (2015).

15 Bouyssié, D., Hesse, A. M., Mouton-Barbosa, E. *et al.* Proline: an efficient and user-friendly software suite for large-scale proteomics. *Bioinformatics (Oxford, England)* **36**, 3148-3155, doi:10.1093/bioinformatics/btaa118 (2020).

16 Schwanhäusser, B., Busse, D., Li, N. *et al.* Global quantification of mammalian gene expression control. *Nature* **473**, 337-342, doi:10.1038/nature10098 (2011).

17 Smits, A. H., Jansen, P. W., Poser, I. *et al.* Stoichiometry of chromatin-associated protein complexes revealed by label-free quantitative mass spectrometry-based proteomics. *Nucleic acids research* **41**, e28, doi:10.1093/nar/gks941 (2013).

18 Gennaris, A., Nguyen, V. S., Thouvenel, L. *et al.* Optimal functioning of the Lpt bridge depends on a ternary complex between the lipocalin YedD and the LptDE translocon. *Cell reports* **44**, 115446, doi:10.1016/j.celrep.2025.115446 (2025).

19 Lomize, M. A., Pogozheva, I. D., Joo, H. *et al.* OPM database and PPM web server: resources for positioning of proteins in membranes. *Nucleic acids research* **40**, D370-376, doi:10.1093/nar/gkr703 (2012).

20 Grenier, F., Matteau, D., Baby, V. *et al.* Complete Genome Sequence of Escherichia coli BW25113. *Genome announcements* **2**, doi:10.1128/genomeA.01038-14 (2014).

21 Ranava, D., Yang, Y., Orenday-Tapia, L. *et al.* Lipoprotein DolP supports proper folding of BamA in the bacterial outer membrane promoting fitness upon envelope stress. *Elife* **10**, doi:10.7554/eLife.67817 (2021).

22 Chin, J. W., Martin, A. B., King, D. S. *et al.* Addition of a photocrosslinking amino acid to the genetic code of Escherichiacoli. *Proceedings of the National Academy of Sciences of the United States of America* **99**, 11020-11024, doi:10.1073/pnas.172226299 (2002).

23 Chaveroche, M. K., Ghigo, J. M. & d'Enfert, C. A rapid method for efficient gene replacement in the filamentous fungus Aspergillus nidulans. *Nucleic acids research* **28**, E97, doi:10.1093/nar/28.22.e97 (2000).
