# Supplementary material for: Structural basis of lipopolysaccharide assembly by the outer membrane translocon holo-complex
Source: Nat Commun. 2025 Nov 24;16:10404. doi: 10.1038/s41467-025-65370-2 (PMC12644819; doi:10.1038/s41467-025-65370-2)
Supplement: Supplementary file 2 — Description of Additional Supplementary Files [file 41467_2025_65370_MOESM2_ESM.pdf]

Description of Additional Supplementary Files

File name: Supplementary Data 1
Description: Amino acid sequences of YedD (LptY) used to build the tree in Supplementary Figure 3.

File name: Supplementary Movie 1
Description: Molecular Dynamics simulations of the Extended and Contracted state complexes of the Lpt system, each over three repeats of 500 ns. The movie illustrates hydrogen bonds between the β1-, β2-, and β26-strands of the barrel, shown as black dashed lines. The amino acid backbones of these three strands are coloured dark grey and displayed as sticks using a CPK colour scheme.
