# Supplementary material for: Structural basis of lipopolysaccharide assembly by the outer membrane translocon holo-complex
Source: Nat Commun. 2025 Nov 24;16:10404. doi: 10.1038/s41467-025-65370-2 (PMC12644819; doi:10.1038/s41467-025-65370-2)
Supplement: Supplementary file 5 — Reporting Summary [file 41467_2025_65370_MOESM5_ESM.pdf]

nature portfolio

Corresponding author(s): Raffaele Ieva
Rémi Fronzes
Phillip Stansfeld

Last updated by author(s): Apr 9, 2025

Reporting Summary

Nature Portfolio wishes to improve the reproducibility of the work that we publish. This form provides structure for consistency and transparency in reporting. For further information on Nature Portfolio policies, see our Editorial Policies and the Editorial Policy Checklist.

Statistics

For all statistical analyses, confirm that the following items are present in the figure legend, table legend, main text, or Methods section.

n/a	Confirmed	
☐	☒	The exact sample size (*n*) for each experimental group/condition, given as a discrete number and unit of measurement
☐	☒	A statement on whether measurements were taken from distinct samples or whether the same sample was measured repeatedly
☐	☒	The statistical test(s) used AND whether they are one- or two-sided<br>*Only common tests should be described solely by name; describe more complex techniques in the Methods section.*
☒	☐	A description of all covariates tested
☒	☐	A description of any assumptions or corrections, such as tests of normality and adjustment for multiple comparisons
☐	☒	A full description of the statistical parameters including central tendency (e.g. means) or other basic estimates (e.g. regression coefficient) AND variation (e.g. standard deviation) or associated estimates of uncertainty (e.g. confidence intervals)
☐	☒	For null hypothesis testing, the test statistic (e.g. *F*, *t*, *r*) with confidence intervals, effect sizes, degrees of freedom and *P* value noted<br>*Give P values as exact values whenever suitable.*
☒	☐	For Bayesian analysis, information on the choice of priors and Markov chain Monte Carlo settings
☒	☐	For hierarchical and complex designs, identification of the appropriate level for tests and full reporting of outcomes
☒	☐	Estimates of effect sizes (e.g. Cohen's *d*, Pearson's *r*), indicating how they were calculated

Our web collection on statistics for biologists contains articles on many of the points above.

Software and code

Policy information about availability of computer code

Data collection	Chemiluminescence signals were acquired using a Bio-Rad ChemiDoc Imaging System and software.<br>Native MS data were acquired on a Synapt G2Si (Waters, Manchester, UK) coupled to an automated chip-based nano-electrospray source (Triversa Nanomate, Advion Biosciences, Ithaca, NY, USA) and LC-MS/MS samples were acquired using a Q- Exactive Plus mass spectrometer (Thermo Fisher Scientific, Bremen, Germany) coupled to an Ultimate 3000 nanoRS system. Cryo-EM data collection was performed on a Titan Krios G4 microscope equipped with a cold field emission gun (C-FEG) operated at 300 kV. Images were recorded using a Falcon 4i direct electron detector in counting mode, coupled to a SelectrisX energy filter with a 10 eV energy slit width. All software tools (including version number and relevant settings) and statistical tests that have been used and citations of the corresponding references are reported in the main text.
Data analysis	The complete list of software used for data analysis includes:<br>Bio-Rad Image Lab™ Software (Version 6.0.0 build 25)<br>Prokka (version 1.14.6)<br>OrthoFinder (version 2.5.4)

mafft (v7.520)
IQ-TREE2 (version 2.1.4-beta)
ModelFinder from IQ-TREE2
iTOL [https://itol.embl.de/]
hmmsearch , hmmscan , esl-translate programs from HMMER (package version 3.1b2)
Pfam library (version 34.0)
BlastP and BlastX software from NCBI web server [https://blast.ncbi.nlm.nih.gov/Blast.cgi]
trimAl
SerialEM (version 4.1.0beta)
Digital Micrograph (version 3.6)
cryoSPARC (version 4.4)
AlphaFold Multimer (version 1.0)
ChimeraX (version 1.8)
ISOLDE (version 1.6.0)
COOT (version 1.1.09)
DeepEMhancer (version 0.15)
PHENIX (version 1.21)
MolProbity (version 4.5.2)
Gromacs (version 2024)
PyMOL (version 3)
MDAnalysis (version 2.8)
MassLynX (version 4.1)
pwiz-mzdb converter (version 0.9.10)
mzdb-access library (version 0.7)
Mascot search engine (version 2.8.3)
Proline (version 2.1)

For manuscripts utilizing custom algorithms or software that are central to the research but not yet described in published literature, software must be made available to editors and reviewers. We strongly encourage code deposition in a community repository (e.g. GitHub). See the Nature Portfolio guidelines for submitting code & software for further information.

Data

Policy information about availability of data

All manuscripts must include a data availability statement. This statement should provide the following information, where applicable:

- Accession codes, unique identifiers, or web links for publicly available datasets
- A description of any restrictions on data availability
- For clinical datasets or third party data, please ensure that the statement adheres to our policy

The following Data Availability statement is provided within the manuscript.
LptDE model information is available under accession PDB 9I9Z and the density map under accession EMDB 52773. LptDEY map is available under EMDB 52777 and the pertaining LptDE model under accession PDB 9IA0. LptDEM model information is available under accession PDB 9IA2 and the density map under accession EMDB 52778. LptDEMY map is available under EMDB 52779 and the pertaining LptDEM model under accession PDB 9IA5. MD simulations source data are available at the Zenodo repository, [https://doi.org/10.5281/zenodo.16643358]. Source data relative to the native MS analysis have been deposited to the Pride partner repository with the dataset identifier PXD041774 and PXD068376, [http://proteomecentral.proteomexchange.org/cgi/GetDataset?ID=PXD041774] and [http://proteomecentral.proteomexchange.org/cgi/GetDataset?ID=PXD068376]. LC-MS/MS source data have been deposited to the Pride partner repository with the dataset identifier PXD068376, [http://proteomecentral.proteomexchange.org/cgi/GetDataset?ID=PXD068376]. Other source data are provided as a Source Data file. These include source data used for the phylogenetic analysis, source data relative to the LC-MS/MS analysis in Supplementary Fig. 1b and to the densitometry quantifications of Fig. 5e, uncropped scans of all blots and gels in Figures. Uncropped scans of all blots and gels in Supplementary Figures are supplied in Supplementary Fig. 19.

Research involving human participants, their data, or biological material

Policy information about studies with human participants or human data. See also policy information about sex, gender (identity/presentation), and sexual orientation and race, ethnicity and racism.

Reporting on sex and gender	n/a
Reporting on race, ethnicity, or other socially relevant groupings	n/a
Population characteristics	n/a
Recruitment	n/a
Ethics oversight	n/a

Note that full information on the approval of the study protocol must also be provided in the manuscript.

Field-specific reporting

Please select the one below that is the best fit for your research. If you are not sure, read the appropriate sections before making your selection.

☒ Life sciences ☐ Behavioural & social sciences ☐ Ecological, evolutionary & environmental sciences

For a reference copy of the document with all sections, see nature.com/documents/nr-reporting-summary-flat.pdf

Life sciences study design

All studies must disclose on these points even when the disclosure is negative.

Sample size	No sample size determination was needed. The number of independent experiments was determined based on the level of variability within replicates and based on the experience with each methodology. Independent triplicates (indicated in Figure Legends and Methods) were performed for each experiment as standards for the techniques used
Data exclusions	No data exclusion was needed.
Replication	Each experiment was repeated at least three times. All attempts at replication were successful. In the case of protein gels, representative results are shown.
Randomization	Randomization is not relevant to our analyses as there were no covariates applicable within this study.
Blinding	Blinding was not required, as it is not necessary for the techniques used.

Reporting for specific materials, systems and methods

We require information from authors about some types of materials, experimental systems and methods used in many studies. Here, indicate whether each material, system or method listed is relevant to your study. If you are not sure if a list item applies to your research, read the appropriate section before selecting a response.

Materials & experimental systems

n/a	Involved in the study	
☐	☒	Antibodies
☒	☐	Eukaryotic cell lines
☒	☐	Palaeontology and archaeology
☒	☐	Animals and other organisms
☒	☐	Clinical data
☒	☐	Dual use research of concern
☒	☐	Plants

Methods

n/a	Involved in the study	
☒	☐	ChIP-seq
☒	☐	Flow cytometry
☒	☐	MRI-based neuroimaging

Antibodies

Antibodies used	Antisera were raised in rabbits against peptides or full proteins from Escherichia coli (LptM, dilution 1:1000; BamA 1:1000; BamD 1:1000; BamE 1:1000; SurA 1:1000; LamB 1:1000; LptY 1:1000; LptD 1:5000; LptE 1:5000; LptA 1:1000). Horseraddish peroxidase conjugated anti-rabbit IgG (Sigma A6154 1:10000), anti LPS monoclonal antibody (Hycult WN1 222-5) and Horseraddish peroxidase conjugated anti-polyHistidine antibodies (TaKaRa n. 631210 1:2000) were purchased.
Validation	Each antibody was tested for specificity by SDS-PAGE and western blotting, comparing the signals obtained from cell lysates of E. coli and deletion strains or strains extpressing truncated or tagged variants of the proteins of interest. Absence or shifts of protein signals proved specificity.

April 2023

Plants

Seed stocks	*Report on the source of all seed stocks or other plant material used. If applicable, state the seed stock centre and catalogue number. If plant specimens were collected from the field, describe the collection location, date and sampling procedures.*
Novel plant genotypes	*Describe the methods by which all novel plant genotypes were produced. This includes those generated by transgenic approaches, gene editing, chemical/radiation-based mutagenesis and hybridization. For transgenic lines, describe the transformation method, the number of independent lines analyzed and the generation upon which experiments were performed. For gene-edited lines, describe the editor used, the endogenous sequence targeted for editing, the targeting guide RNA sequence (if applicable) and how the editor was applied.*
Authentication	*Describe any authentication procedures for each seed stock used or novel genotype generated. Describe any experiments used to assess the effect of a mutation and, where applicable, how potential secondary effects (e.g. second site T-DNA insertions, mosiacism, off-target gene editing) were examined.*
